# Supplementary material for: The hidden hyperbolic geometry of international trade: World Trade Atlas 1870–2013
Source: Sci Rep. 2016 Sep 16;6:33441. doi: 10.1038/srep33441 (PMC5025783; doi:10.1038/srep33441)
Supplement: Supplementary Information [file srep33441-s7.pdf]

Supplementary Information for
"The hidden hyperbolic geometry of international trade:
World Trade Atlas 1870-2013"

Guillermo García-Pérez,[1,2] Marián Boguñá,[1,2] Antoine Allard,[1,2] and M. Ángeles Serrano[1,3,2]

[1]*Departament de Física de la Matèria Condensada, Universitat de Barcelona, Martí i Franquès 1, 08028 Barcelona, Spain*
[2]*Universitat de Barcelona Institute of Complex Systems (UBICS), Universitat de Barcelona, Barcelona, Spain*
[3]*ICREA, Pg. Lluís Companys 23, E-08010 Barcelona, Spain*

Contents

I. Empirical data 1

II. Methods 4
A. Traits of globalization in the reconstructed trade networks 5
B. Backbone construction 5
C. Embedding into hyperbolic space 11
D. Community detection 26
E. Preferential Trade Agreements 28

References 30

I. EMPIRICAL DATA

In order to study the evolution of the World Trade Web (WTW), we have constructed a network for every year in which nodes are countries and the links between them carry a weight ω_{ij} which is equal to the sum of the trade flows from i to j and from j to i, i. e. $\omega_{ij} = \omega_{i\to j} + \omega_{j\to i}$. Hence, the resulting networks are positively weighted and undirected. We now describe how we obtained the bilateral trade data needed to generate the networks.

The Correlates of War Project (COW) (Refs. [26, 27] in the paper) provides a database with the bilateral trade flows from 1870 to 2009, as well as a detailed document with the procedure followed to obtain the data. We have used this data from 1870 to 1996 (we have removed the flows between Haiti and United Kingdom in 1877, which seem to be an outliar). However, since we detected some flaws in the dataset for the last years—like the absence of trade data for Germany after 2006—and we wanted to complement the dataset with recent data, we followed the same steps to reconstruct the bilateral trade data from 1997 to 2013. The first step in this process is to download the trade data from the International Monetary Fund (IMF) [1]. This database includes the trade data as reported by each country. Thus, a given country may appear under different names—for instance, some countries still report trade with the U.S.S.R. To solve this, we first need to match the different countries appearing in the dataset with the COW's state list, which we have extended from 2011 to 2013 (COW's country codes are collected in Table S1). In most cases, the matching is very clear, but there are some special cases: China's trade data does not include that of the two special administrative regions Hong Kong and Macao. We have added those flows to China. Also, there are many entries that correspond to "Yugoslavia" in the COW's state list ("Yugoslavia", "Yugoslavia not specified", "Serbia and Montenegro", "Serbia and Montenegro not specified", "Serbia, Republic of", "Montenegro" and "Kosovo" before its independence). The flows to or from "U.S.S.R. not specified" have been redirected to "Russia" and the trade of "South Sudan" in 2011 to "Sudan". Also, several countries report trade with "Belgium-Luxembourg not specified"; following the same steps as the COW Project, we have split every such flow into two, proportionally to the GDP of Belgium and Luxembourg the corresponding year. The GDP data of both countries for this operation was retrieved from the World Bank [2]. Finally, there are no trade reports from Taiwan in the IMF database. We have obtained its data from the ROC's Bureau of Foreign Trade [3]. Again, we have matched their country list with the one from the COW Project.

Num. code	3 letter code	Name	Num. code	3 letter code	Name	Num. code	3 letter code	Name
2	USA	United States of America	338	MLT	Malta	580	MAG	Madagascar
20	CAN	Canada	339	ALB	Albania	581	COM	Comoros
31	BHM	Bahamas	341	MNG	Montenegro	590	MAS	Mauritius
40	CUB	Cuba	343	MAC	Macedonia	591	SEY	Seychelles
41	HAI	Haiti	344	CRO	Croatia	600	MOR	Morocco
42	DOM	Dominican Republic	345	YUG	Yugoslavia	615	ALG	Algeria
51	JAM	Jamaica	346	BOS	Bosnia and Herzegovina	616	TUN	Tunisia
52	TRI	Trinidad and Tobago	347	KOS	Kosovo	620	LIB	Libya
53	BAR	Barbados	349	SLV	Slovenia	625	SUD	Sudan
54	DMA	Dominica	350	GRC	Greece	626	SSD	South Sudan
55	GRN	Grenada	352	CYP	Cyprus	630	IRN	Iran
56	SLU	St. Lucia	355	BUL	Bulgaria	640	TUR	Turkey
57	SVG	St. Vincent and the Grenadines	359	MLD	Moldova	645	IRQ	Iraq
58	AAB	Antigua & Barbuda	360	ROM	Romania	651	EGY	Egypt
60	SKN	St. Kitts and Nevis	365	RUS	Russia	652	SYR	Syria
70	MEX	Mexico	366	EST	Estonia	660	LEB	Lebanon
80	BLZ	Belize	367	LAT	Latvia	663	JOR	Jordan
90	GUA	Guatemala	368	LIT	Lithuania	666	ISR	Israel
91	HON	Honduras	369	UKR	Ukraine	670	SAU	Saudi Arabia
92	SAL	El Salvador	370	BLR	Belarus	678	YAR	Yemen Arab Republic
93	NIC	Nicaragua	371	ARM	Armenia	679	YEM	Yemen
94	COS	Costa Rica	372	GRG	Georgia	680	YPR	Yemen People's Republic
95	PAN	Panama	373	AZE	Azerbaijan	690	KUW	Kuwait
100	COL	Colombia	375	FIN	Finland	692	BAH	Bahrain
101	VEN	Venezuela	380	SWD	Sweden	694	QAT	Qatar
110	GUY	Guyana	385	NOR	Norway	696	UAE	United Arab Emirates
115	SUR	Suriname	390	DEN	Denmark	698	OMA	Oman
130	ECU	Ecuador	395	ICE	Iceland	700	AFG	Afghanistan
135	PER	Peru	402	CAP	Cape Verde	701	TKM	Turkmenistan
140	BRA	Brazil	403	STP	Sao Tome and Principe	702	TAJ	Tajikistan
145	BOL	Bolivia	404	GNB	Guinea-Bissau	703	KYR	Kyrgyzstan
150	PAR	Paraguay	411	EQG	Equatorial Guinea	704	UZB	Uzbekistan
155	CHL	Chile	420	GAM	Gambia	705	KZK	Kazakhstan
160	ARG	Argentina	432	MLI	Mali	710	CHN	China
165	URU	Uruguay	433	SEN	Senegal	712	MON	Mongolia
200	UKG	United Kingdom	434	BEN	Benin	713	TAW	Taiwan
205	IRE	Ireland	435	MAA	Mauritania	730	KOR	Korea
210	NTH	Netherlands	436	NIR	Niger	731	PRK	North Korea
211	BEL	Belgium	437	CDI	Ivory Coast	732	ROK	South Korea
212	LUX	Luxembourg	438	GUI	Guinea	740	JPN	Japan
220	FRN	France	439	BFO	Burkina Faso	750	IND	India
221	MNC	Monaco	450	LBR	Liberia	760	BHU	Bhutan
223	LIE	Liechtenstein	451	SIE	Sierra Leone	770	PAK	Pakistan
225	SWZ	Switzerland	452	GHA	Ghana	771	BNG	Bangladesh
230	SPN	Spain	461	TOG	Togo	775	MYA	Myanmar
232	AND	Andorra	471	CAO	Cameroon	780	SRI	Sri Lanka
235	POR	Portugal	475	NIG	Nigeria	781	MAD	Maldives
240	HAN	Hanover	481	GAB	Gabon	790	NEP	Nepal
245	BAV	Bavaria	482	CEN	Central African Republic	800	THI	Thailand
255	GMY	Germany	483	CHA	Chad	811	CAM	Cambodia
260	GFR	German Federal Republic	484	CON	Congo	812	LAO	Laos
265	GDR	German Democratic Republic	490	DRC	Democratic Republic of the Congo	816	DRV	Vietnam
267	BAD	Baden	500	UGA	Uganda	817	RVN	Republic of Vietnam
269	SAX	Saxony	501	KEN	Kenya	820	MAL	Malaysia
271	WRT	Wuerttemburg	510	TAZ	Tanzania	830	SIN	Singapore
273	HSE	Hesse Electoral	511	ZAN	Zanzibar	835	BRU	Brunei
275	HSG	Hesse Grand Ducal	516	BUI	Burundi	840	PHI	Philippines
280	MEC	Mecklenburg Schwerin	517	RWA	Rwanda	850	INS	Indonesia
290	POL	Poland	520	SOM	Somalia	860	ETM	East Timor
300	AUH	Austria-Hungary	522	DJI	Djibouti	900	AUL	Australia
305	AUS	Austria	530	ETH	Ethiopia	910	PNG	Papua New Guinea
310	HUN	Hungary	531	ERI	Eritrea	920	NEW	New Zealand
315	CZE	Czechoslovakia	540	ANG	Angola	935	VAN	Vanuatu
316	CZR	Czech Republic	541	MZM	Mozambique	940	SOL	Solomon Islands
317	SLO	Slovakia	551	ZAM	Zambia	946	KIR	Kiribati
325	ITA	Italy	552	ZIM	Zimbabwe	947	TUV	Tuvalu
327	PAP	Papal States	553	MAW	Malawi	950	FIJ	Fiji
329	SIC	Two Sicilies	560	SAF	South Africa	955	TON	Tonga
331	SNM	San Marino	565	NAM	Namibia	970	NAU	Nauru
332	MOD	Modena	570	LES	Lesotho	983	MSI	Marshall Islands
335	PMA	Parma	571	BOT	Botswana	986	PAL	Palau
337	TUS	Tuscany	572	SWA	Swaziland	987	FSM	Federated States of Micronesia
						990	WSM	Samoa

TABLE S1: COW's state list. Every country is identified by a numeric code and a three-letter code.

Once the correspondence between databases was established, we were able to reconstruct the trade flows. In the database, there were four numbers between any two countries i and j: $\omega^i_{i\to j}$, $\omega^j_{i\to j}$, $\omega^i_{j\to i}$ and $\omega^j_{j\to i}$, where the upper index represents the reporting country and the arrow represents the direction of the flow of goods. According to the COW Project, the importer's value is generally more reliable than the exporter's—due to the taxes to which imports are subject—, so we have used the values of $\omega^j_{i\to j}$ and $\omega^i_{j\to i}$ whenever they were available. If these values were not reported, we used the exporters' values $\omega^i_{i\to j}$ and $\omega^j_{j\to i}$ instead. In Fig. S1 we compare the trade flows of 1997 in both databases, DBI and DBII. In most cases, the flows are equal. Indeed, the difference between values is smaller than 10^{-2} US Million \$ in the 96.1% of cases, which indicates a very good agreement (notice also that there is no abrupt change in any of the results between 1996 and 1997 despite the change of database). The few differences are due to rounding criteria in some cases—we round to the second decimal place if the flow is larger than 1 US Million \$, whereas there appear to be different rounding criteria in DBI—and to differences in the original IMF datasets in others—the differences between the importers' and the exporters' reported values is very large in some cases (for instance, $\omega^{SLV}_{SLV\to ZIM} \approx 160000$ US \$ while $\omega^{ZIM}_{SLV\to ZIM} \approx 15$ US \$), so the biggest differences between both datasets are probably caused by the absence of some importers' reports when DBI was constructed. Nonetheless, as stated above, these cases are very rare.

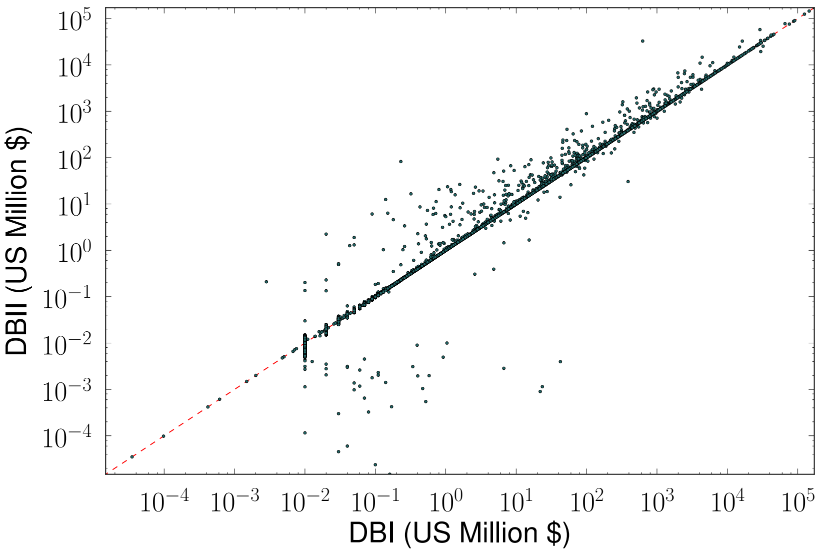

FIG. S1: Comparison between DBI and DBII in 1997. Each point corresponds to the values of a common trade flow in both databases. Although some dispersion can be observed, most points lay in the diagonal (the difference between both values is less than 10^{-2} US Million \$ in the 96.1% of cases). Indeed, there is no abrupt change in any of the results between 1996 and 1997 despite the change of database.

The GDP data were obtained from three different sources: The Maddison Project [4] from 1870 to 1949, Gleditsch's GDP data [5] from 1950 to 2011 and data from the World Bank and the CIA World Factbook [6] for years 2012 and 2013. As in the case of the trade data, we needed to match the countries in these databases with the COW's state list. The Maddison Project provides GDP data with a fixed country list, so the two country lists were very different. We thus provide a detailed table (Table S2) in which we show how the GDPs of all datasets have been distributed among the countries in our networks. Moreover, since trade is reported in current US Million dollars, we have converted the real GDP values into current GDP values using the deflator calculated by comparing Maddison's data with data from [7].

Country in dataset	COW state	Period	Country in dataset	COW state	Period
Yugoslavia	Turkey	1870 - 1877	Malaysia	Japan	1942 - 1956
Romania	Turkey	1870 - 1877	Singapore	UK	1870 - 1941
Tunisia	France	1882 - 1955	Singapore	Japan	1942 - 1964
Egypt	UK	1883 - 1936	Jordan	UK	1870 - 1945
North Korea	China	1870 - 1886	India	UK	1870 - 1946
South Korea	China	1870 - 1886	Myanmar	UK	1870 - 1947
North Korea	Korea	1887 - 1905	Sri Lanka	UK	1870 - 1947
South Korea	Korea	1887 - 1905	Taiwan	China	1870 - 1948
North Korea	Japan	1906 - 1948	Indonesia	Netherlands	1870 - 1948
South Korea	Japan	1906 - 1949	Vietnam	France	1870 - 1953
Philippines	Spain	1870 - 1897	Ghana	UK	1870 - 1956
Philippines	USA	1898 - 1945	Syria	Egypt	1959 - 1960
Norway	Sweden	1870 - 1904	Jamaica	UK	1870 - 1961
Bulgaria	Turkey	1870 - 1907	Algeria	France	1870 - 1961
Morocco	France	1913 - 1955	Bangladesh	Pakistan	1947 - 1970
Albania	Turkey	1870 - 1913	Oman	Uk	1950 - 1970
Samoa	Germany	1900 - 1914	Bhutan	India	1950 - 1970
Samoa	New Zealand	1915 - 1962	Marshall Islands	USA	1986 - 1990
Finland	Russia	1870 - 1916	Micronesia	USA	1986 - 1990
Czechoslovakia	Austria-Hungary	1870 - 1917	Tonga	UK	1970 - 1998
Poland	Austria-Hungary, Russia, Germany	1870 - 1917	Kiribati	UK	1979 - 1998
Austria	Austria-Hungary	1870 - 1918	Tuvalu	UK	1978 - 1999
Hungary	Austria-Hungary	1870 - 1918	Macedonia	Yugoslavia	1992
Canada	UK	1870 - 1919	Croatia	Yugoslavia	1991
South Africa	UK	1870 - 1919	Serbia	Yugoslavia	2006 - 2013
Australia	UK	1870 - 1919	Abkhazia	Georgia	2008 - 2011
New Zealand	UK	1870 - 1919	South Ossetia	Georgia	2008 - 2011
Syria	Turkey	1870 - 1919	Montenegro	Yugoslavia	2006
Lebanon	Turkey	1870 - 1919	Kosovo	Yugoslavia	2008
Syria	France	1920 - 1945	German Federal Republic	Germany	1991 - 2011
Lebanon	France	1920 - 1945	Arab Republic of Yemen	Yemen	1990 - 2011
Ireland	UK	1870 - 1921	People's Republic of Yemen	Yemen	1990
Iraq	UK	1870 - 1931	Macao	China	2012 - 2013
Malaysia	UK	1870 - 1941	Hong Kong	China	2012 - 2013

TABLE S2: Assignments of GDPs between different datasets. The left column corresponds to the county in the original dataset (Maddison Project, Gleditsch's GDP data, World Bank or CIA Factbook). The right column shows the state in the COW's state list to which the GDP value has been assigned in the period displayed in the third column. In the case of Poland between 1870 – 1917, we have assigned a third of its GDP to each country in the second column.

The PTAs data was obtained from the World Trade Organization (WTO) (Ref. [41] in the paper). Again, we matched the names of countries with the ones in our data.

II. METHODS

In this section we give a detailed description of the methods used in this work. We first show some trends in the reconstructed trade networks that are related to globalization (Section II A), we then explain how we filtered the networks to obtain the backbones (Section II B), how we embedded the backbones into hyperbolic space (Section II C) and, finally, in Section II D we describe the Critical Gap Method (CGM) for community detection, which relies on the geometrical information obtained from the hyperbolic embedding.

A. Traits of globalization in the reconstructed trade networks

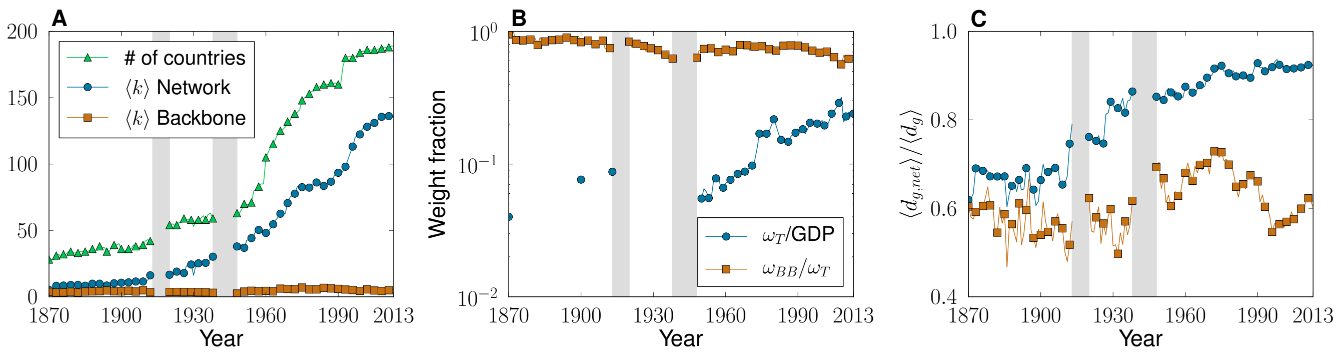

FIG. S2: Comparison between the properties of the original world trade network and the corresponding backbone extracted from it for the different years. **A**: Evolution of the number of states and the average number of trade partners per country, $\langle k \rangle$, both for the original network and the backbone. **B**: Evolution of the ratio between total trade ω_T and world's GDP. The sparsity of points before the WWII is due to lack of GDP data. The plot also shows the fraction of total weight that is retained in the backbone (70% $\sim$ 80%). **C**: Evolution of the ratio between the average geographic distance of trade channels $\langle d_{g,net} \rangle$ and the average distance among any pair of countries in the world $\langle d_g \rangle$.

The reconstructed trade networks over the past fourteen decades show trends consistent with globalization. The average number of trade partners per country $\langle k \rangle$ increases at a faster rate than that of the number of countries N, Fig. S2**A**. As a consequence, the density of connections $\langle k \rangle/(N-1)$ has boosted from 0.2 to 0.7 in the period under study. The temporal evolution of the total international trade—measured as a percentage of the sum of all national gross domestic products (GDPs)—presents a similar trend, with a major reversal in the interwar periods, Fig. S2**B**. At the same time, the average geographic length of trade channels has been growing as compared to the average distance among all pairs of countries in the system, Fig. S2**C**.

B. Backbone construction

The overall flux organization at the global scale can be characterized by the distribution $P(\omega_{ij})$ denoting the probability that any given connection is carrying a flow ω_{ij}. The observed distribution is heavy-tailed and spans approximately four orders of magnitude (Ref. [11] in the paper). Such a feature implies that only a small percentage of all the connections in the network carry most of its total flow, with most of the flows below the average and some of them with a much higher value. Heterogeneity is also present at the local level; i.e., in the distribution of flows associated to the connections of a given country.

In order to assess the effect of inhomogeneities at the local level, for each country i with k trade partners we calculate the extensively used in economics standard indicator of market concentration referred to as the Herfindahl-Hirschman Index or HHI (Refs. [31, 32] in the paper), also denoted as the disparity measure in the complex networks literature [8],

$$kY_i(k) = k \sum_j \left(\frac{\omega_{ij}}{s_i} \right)^2 , \tag{S1}$$

where ω_{ij} is the total flow between countries i and j and $s_i = \sum_j \omega_{ij}$ is the strength (aggregated trade) of country i. If country i distributes its trade homogeneously between its trade partners, $kY_i(k) = 1$, whereas in the opposite case—if all its trade is concentrated on a single link—, $kY_i(k) = k$. The local heterogeneity in the distribution of trade reveals that not all trade channels are equally significant (see Fig. S3) and thus the disparity filter (Ref. [33] in the paper) can be applied in order to select only trade channels that are significant to at least one of the countries at the end of the channel.

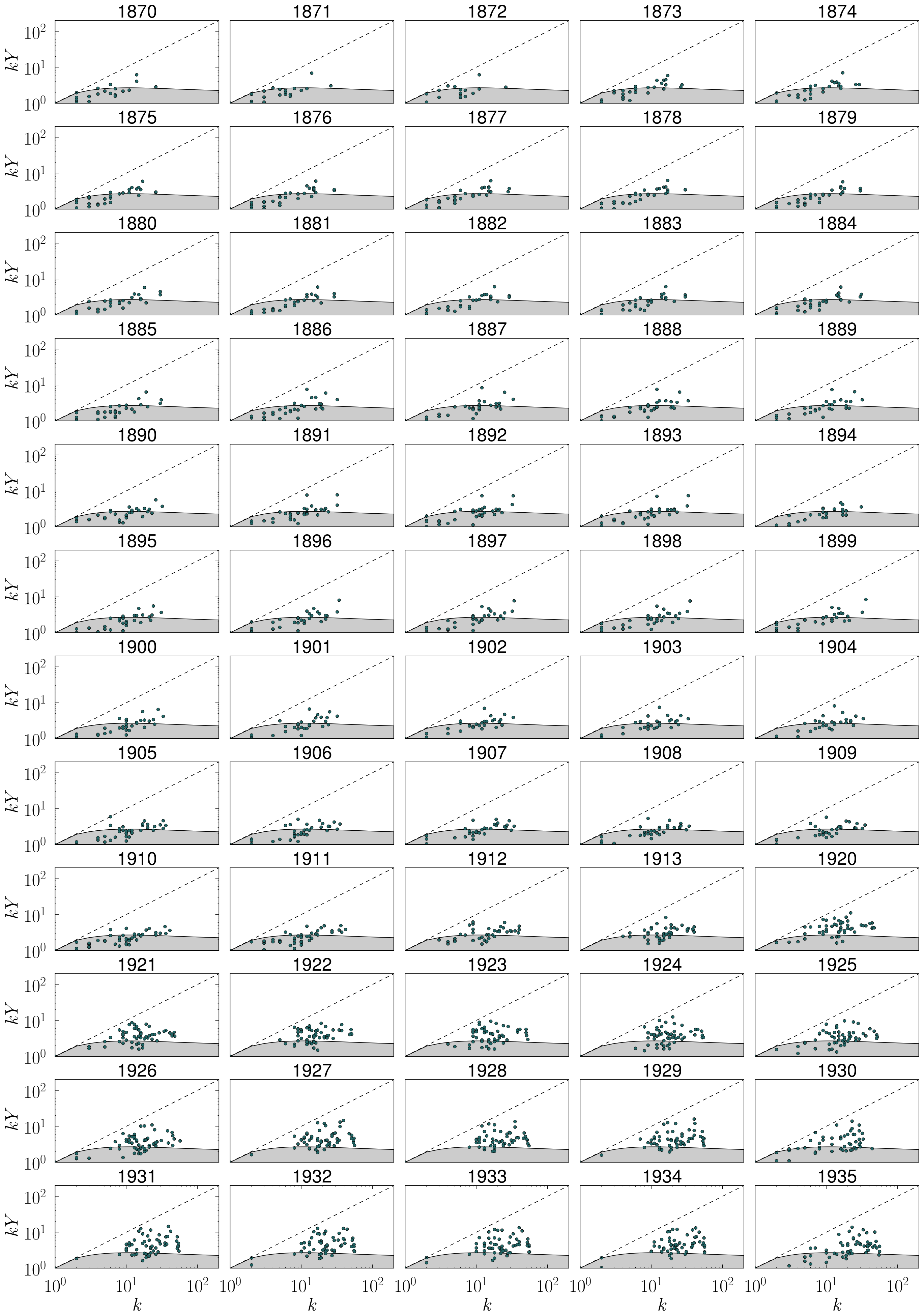
1870
1871
1872
1873
1874
1875
1876
1877
1878
1879
1880
1881
1882
1883
1884
1885
1886
1887
1888
1889
1890
1891
1892
1893
1894
1895
1896
1897
1898
1899
1900
1901
1902
1903
1904
1905
1906
1907
1908
1909
1910
1911
1912
1913
1920
1921
1922
1923
1924
1925
1926
1927
1928
1929
1930
1931
1932
1933
1934
1935
kY
k
10^0
10^1
10^2

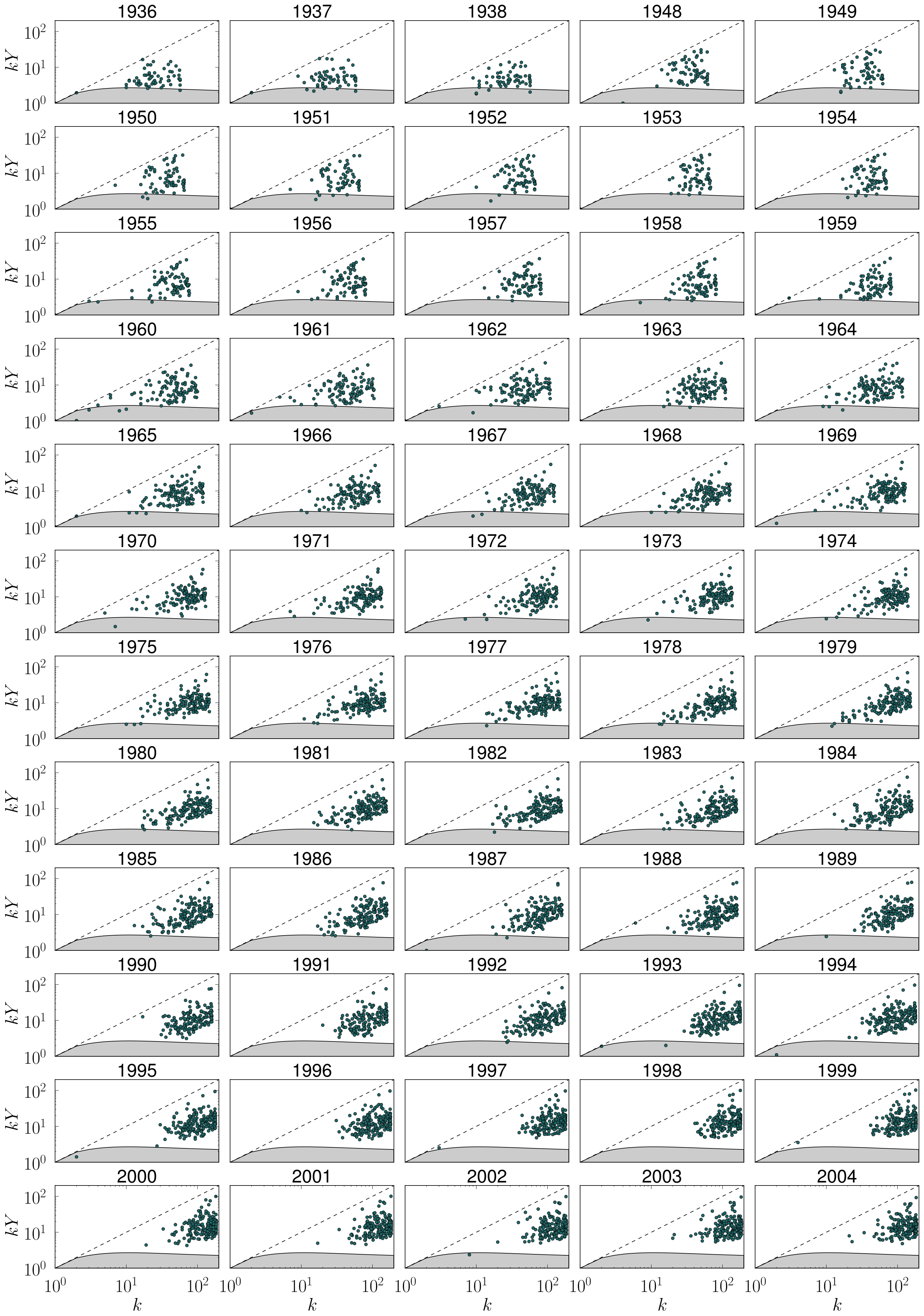
1936
1937
1938
1948
1949
1950
1951
1952
1953
1954
1955
1956
1957
1958
1959
1960
1961
1962
1963
1964
1965
1966
1967
1968
1969
1970
1971
1972
1973
1974
1975
1976
1977
1978
1979
1980
1981
1982
1983
1984
1985
1986
1987
1988
1989
1990
1991
1992
1993
1994
1995
1996
1997
1998
1999
2000
2001
2002
2003
2004
kY
k
10^0
10^1
10^2

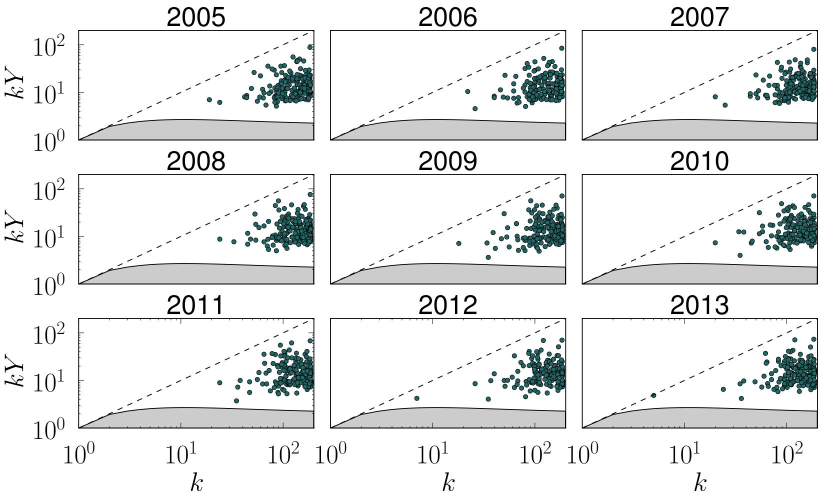

FIG. S3: Evolution of the disparity of all countries in the WTW. Each point in each plot corresponds to a pair $(k_i, kY_i(k))$, where k_i is the degree of country i and $kY_i(k)$ is calculated according to Eq. (S1). The gray area corresponds to the average plus 2 standard deviations given by the null model.

The disparity filter proceeds as follows. For each trade channel of a given country i, we compute the probability α_{ij} that the link takes the observed value ω_{ij}/s_i according to the purely random null model in which countries assign their trade among their partners randomly from a uniform distribution. By imposing a significance level α, we can determine the statistical significance of a given trade channel by comparing α_{ij} to α. Therefore, if $\alpha_{ij} > \alpha$, the flow through that trade channel can be considered compatible with a random distribution (with the chosen significance level α) and is thus discarded. The statistically relevant channels are those that satisfy

$$\alpha_{ij} = 1 - (k-1) \int_0^{\omega_{ij}/s_i} (1-x)^{k-2} dx < \alpha \tag{S2}$$

for at least one of the two countries i and j.

By applying this selection rule to all the links in the network we find the backbone, a new graph containing, in general, less links and nodes. However, the number of links and nodes removed depends on the value of the significance level α. In order to find the appropriate value of α it is convenient to plot the fraction of remaining nodes in the backbone N_{BB}/N vs. the fraction of remaining links L_{BB}/L for different values of α (Fig. S4). As the filter becomes more restrictive, the number of links decreases while keeping almost all nodes until a certain critical point α_c after which the number of nodes starts a steep decay. This behaviour is due to the fact that the disparity filter for $\alpha = \alpha_c$ is able to select the minimal set of significant links that preserve the global connectivity and whose removal results in the isolation of nodes. Geometrically, α_c can be identified with the value of the significance level whose corresponding point in the $N_{BB}/N - L_{BB}/L$ plane maximizes the vertical distance to the diagonal. Notice that on the diagonal $N_{BB}/N = L_{BB}/L$ such that $\langle k \rangle_{BB} = 2L_{BB}/N_{BB} = 2L/N = \langle k \rangle$. Consequently, setting $\alpha = \alpha_c$, where α_c is chosen according to the geometric rule stated above, minimizes the average degree of the backbone $\langle k \rangle_{BB}$. This offers a consistent and systematic criterion to extract the backbones of the World Trade Web at different years. In Fig. S4 we show the curves N_{BB}/N vs. L_{BB}/L as well as the points at which the backbones were extracted. The corresponding values of α_c can be found in Table S4.

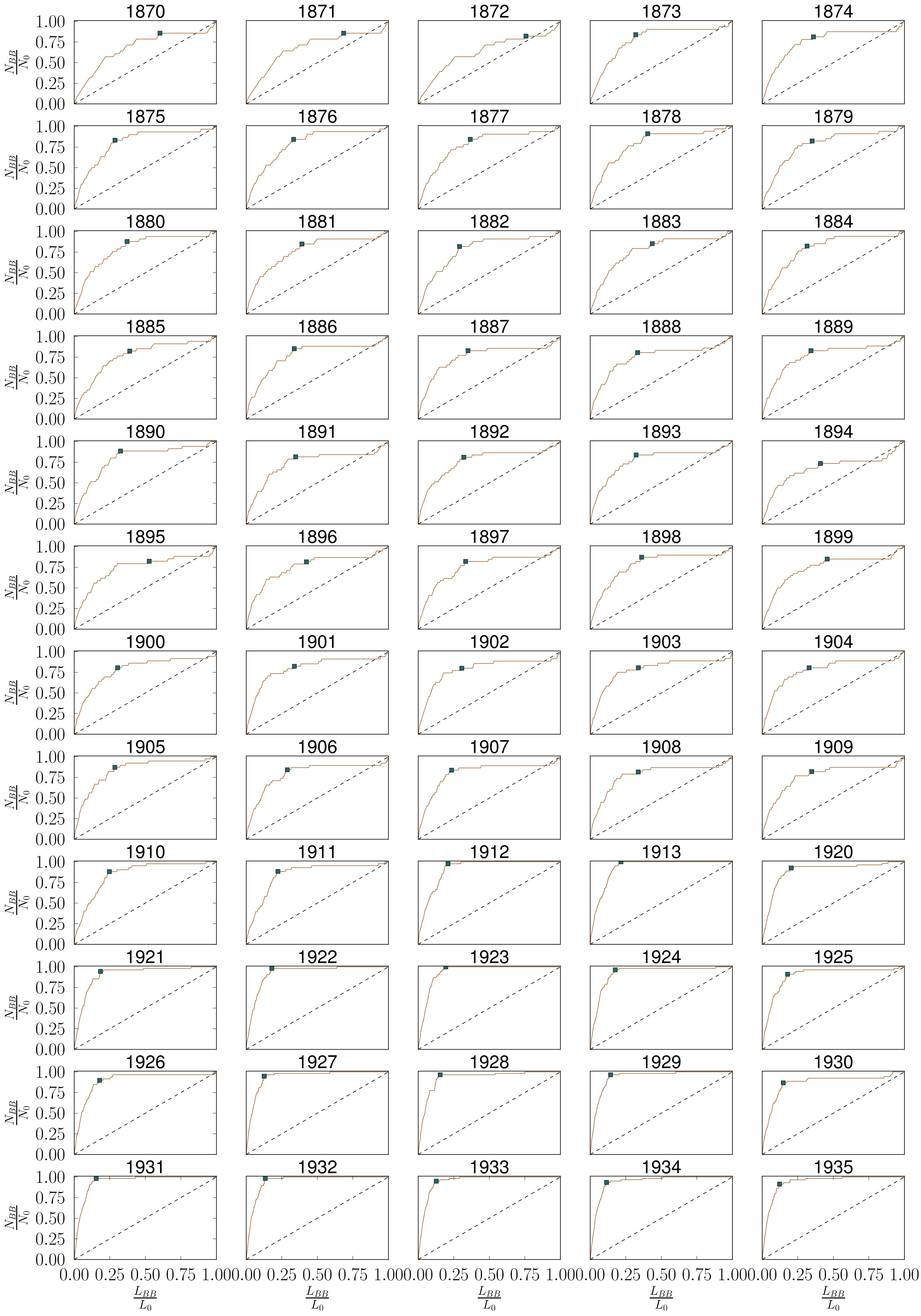

1870
1871
1872
1873
1874
1875
1876
1877
1878
1879
1880
1881
1882
1883
1884
1885
1886
1887
1888
1889
1890
1891
1892
1893
1894
1895
1896
1897
1898
1899
1900
1901
1902
1903
1904
1905
1906
1907
1908
1909
1910
1911
1912
1913
1920
1921
1922
1923
1924
1925
1926
1927
1928
1929
1930
1931
1932
1933
1934
1935
$\frac{N_{BB}}{N_0}$
0.00 0.25 0.50 0.75 1.00
$\frac{L_{BB}}{L_0}$

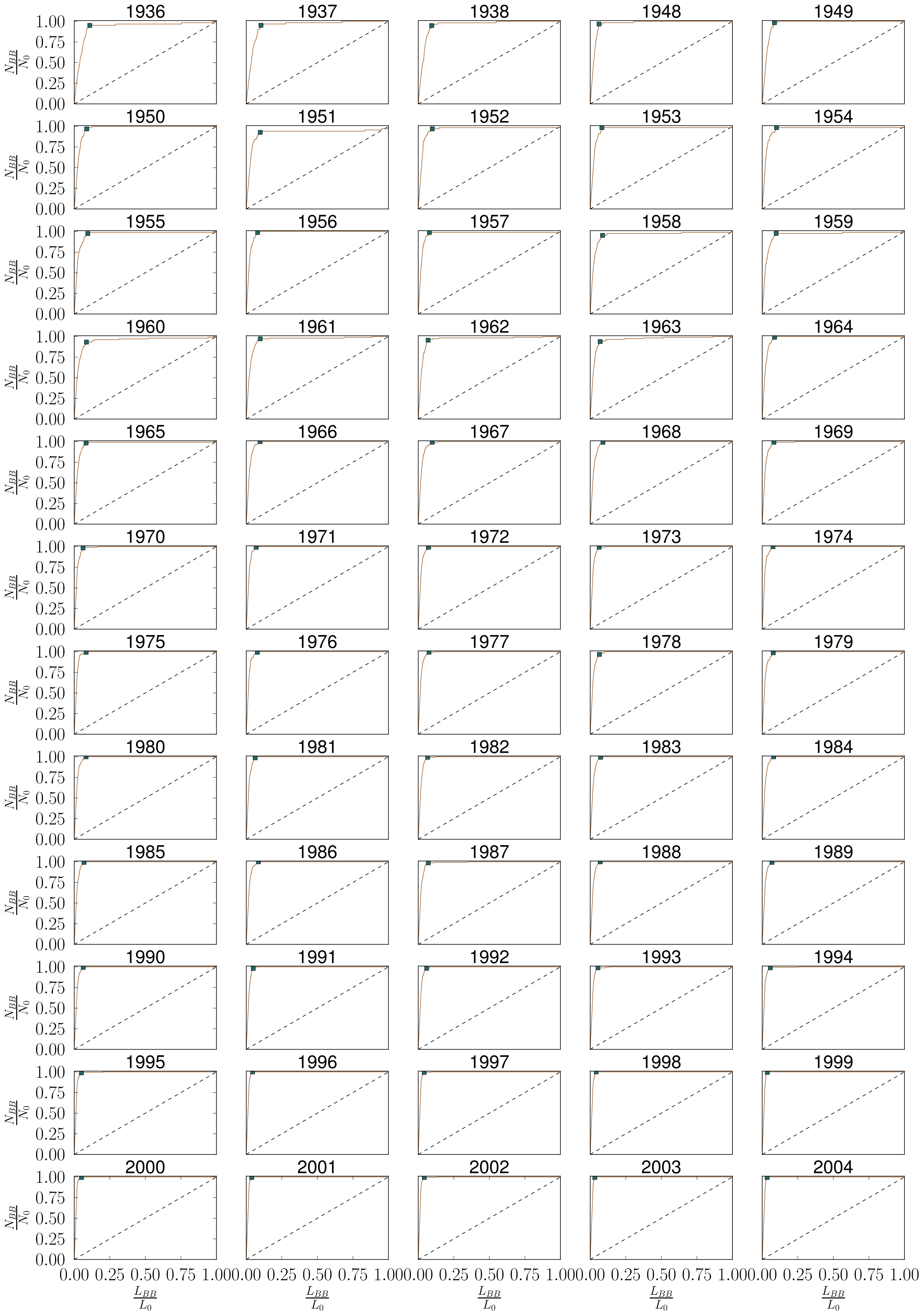
1936
1937
1938
1948
1949
1950
1951
1952
1953
1954
1955
1956
1957
1958
1959
1960
1961
1962
1963
1964
1965
1966
1967
1968
1969
1970
1971
1972
1973
1974
1975
1976
1977
1978
1979
1980
1981
1982
1983
1984
1985
1986
1987
1988
1989
1990
1991
1992
1993
1994
1995
1996
1997
1998
1999
2000
2001
2002
2003
2004
$\frac{N_{BB}}{N_0}$
$\frac{L_{BB}}{L_0}$
0.00
0.25
0.50
0.75
1.00

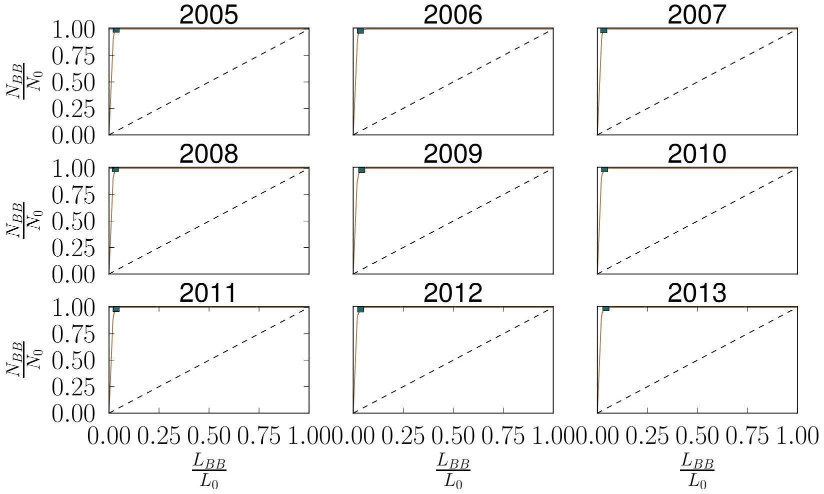

FIG. S4: Curves representing the fraction of nodes N_{BB}/N vs. the fraction of links L_{BB}/L in the backbones parametrized by α. The green squares indicate the values that correspond to the backbone with $\alpha = \alpha_c$ which maximizes the vertical distance to the diagonal (constrained by $N_{BB}/N \geq 0.8$ ($N_{BB}/N \geq 0.7$ in 1894) so that most countries remain in the system. Moreover, in order to avoid fluctuations in the topology between years, the 1975 backbone was selected with a higher link fraction than the given by the geometric rule.)

C. Embedding into hyperbolic space

Hyperbolic geometry has been conjectured as the natural geometry underlying real complex networks (Refs. [21] and [37] in the paper). It is one of the three possible cases of homogeneous and isotropic geometries of constant curvature (in this case negative; the other cases are euclidean and spherical geometries, with zero and positive curvature, respectively). The two pivotal properties of hyperbolic space that make it the natural geometry underlying real complex networks are that it is a metric space, which can induce a non-tree-like topology with clustering (triangles or transitive relations in the graph), and that, due to its negative curvature, space grows exponentially fast with distance. This is homologous to the exponential growth of the number of nodes with diameter observed in small-world networks. It has been proved that random graphs generated from a homogeneous distribution of points in a finite hyperbolic plane have the same topological properties of real complex networks (Ref. [20] in the paper); namely, scale-free degree distributions, strong clustering, the small-world property, etc.

Let us briefly review the hyperbolic model of complex networks (Ref. [20] in the paper) according to which the embedding is performed. In that model, scale-free networks are generated by scattering N nodes randomly into a hyperbolic disk of radius

$$R = 2 \log \left[\frac{2N}{\langle k \rangle \beta \sin \frac{\pi}{\beta}} \left(\frac{\gamma - 1}{\gamma - 2} \right)^2 \right], \tag{S3}$$

where $\langle k \rangle$ is the target average degree of the resulting network, γ is the target exponent of the power-law degree distribution, $P(k) \sim k^{-\gamma}$, and the inverse temperature β is a function of the target average clustering coefficient $\bar{C}$. The distribution of nodes is quasi-uniform, meaning that the angular coordinates $\theta \in [0, 2\pi]$ are distributed uniformly, whereas the radial coordinates $r \in [0, R]$ are distributed with density

$$\rho(r) = \nu e^{\nu(r-R)}, \tag{S4}$$

where $\nu = (\gamma - 1)/2$. Every pair of nodes (i, j) is then connected with a probability that depends on the hyperbolic distance $d_{h,ij}$ between them as

$$p(d_{h,ij}) = \frac{1}{1 + e^{\frac{\beta}{2}(d_{h,ij} - R)}}, \tag{S5}$$

which is the Fermi-Dirac distribution. The distance $d_{h,ij}$ between two nodes with coordinates (r_i, θ_i) and (r_j, θ_j) is given by the hyperbolic law of cosines,

$$\cosh d_{h,ij} = \cosh r_i \cosh r_j - \sinh r_i \sinh r_j \cos d_{a,ij}, \tag{S6}$$

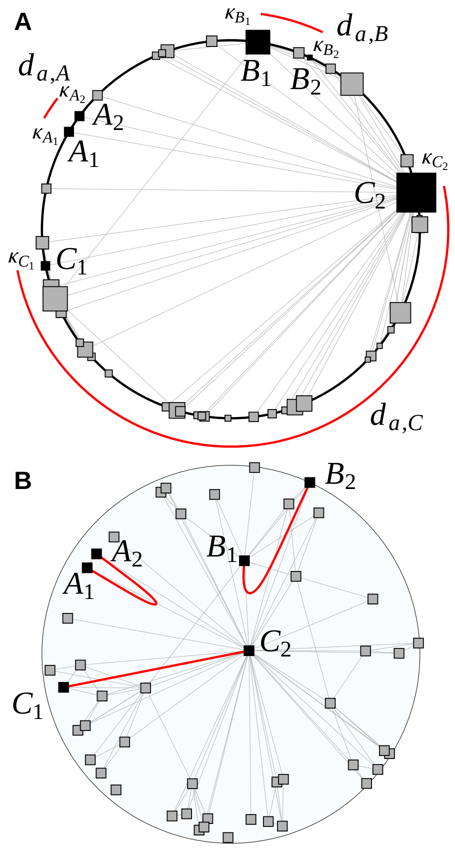

FIG. S5: **Hyperbolic representation of the gravity model.** Three different pairs of nodes $A_1 - A_2$, $B_1 - B_2$ and $C_1 - C_2$ have been highlighted. **A**: Gravity model—Eq. (1) in the paper—in which the effective distance d_a is given by the angular separation in the circle. The size of a node is proportional to its economic size κ. **B**: Hyperbolic space representation of the gravity model of trade channels. The three different pairs of nodes are separated by the same hyperbolic distance. Notice that all nodes are equal size and that nodes with higher degree (larger size in **A**) are positioned closer to the centre in the hyperbolic plane. Nodes at the same hyperbolic distance can be separated by different angular distances depending on their degrees (sizes). As an example, nodes $A_1 - A_2$ with small degree and very low angular separation can be far apart in hyperbolic space.

where $d_{a,ij} \equiv \min(|\Delta\theta_{ij}|, 2\pi - |\Delta\theta_{ij}|)$. The angular separation in hyperbolic space still corresponds to the effective distance in the gravity model of trade channels and it is the radial component which captures the contribution of economic size. The advantage of this approach is that it condensates in a pure geometric framework the properties of the entire system—economic size and effective distance in the gravity model, Eq. (1) in the paper; that is, it allows to draw genuine maps of the trade system where different parts can be compared on an equal footing. More specifically, due to the inverse relation between economic size κ and hyperbolic radius r, large economies with high degrees are located close to the center of the disk, whereas small economies are placed near its boundary. The comparison of distances also needs of some care. While the hyperbolic distance between the origin of coordinates and any point at radial coordinate r is simply r, hyperbolic distances between points with angular separations are more involved. The expression for the approximated hyperbolic distance between any two countries i and j, x_{ij}, introduced in the paper gives us some clues to interpret it. Such distance is roughly the sum of the distances between the two points and the origin of coordinates minus a term that depends on the angular separation between them. This means that the geodesic (or shortest) path connecting i and j first approaches the center of the disk, it bends until it reaches the angular coordinate of j and then moves away from the origin until j is reached. This is illustrated in Fig. S5, where several hyperbolic distances of the same magnitude are plotted. Note that countries in the hyperbolic plane representation are all equal size since all the factors determining their likelihood to form trade channels—angular trade distances and economic sizes in our gravity model—are combined into hyperbolic distances.

This model can also be used to embed networks into hyperbolic space, i.e., to build maps in which distances depend purely on the observed connections between nodes. To do so, we reverse the network generation process: instead

of generating a network from the coordinates of nodes, we need to find the positions of nodes given the resulting network. The way to proceed is to find the coordinates of every country such that the likelihood that the observed WTW is generated by the model described above is maximal. The likelihood is given by

$$L = \prod_{i<j} \left[p(d_{h,ij})\right]^{a_{ij}} \left[1 - p(d_{h,ij})\right]^{1-a_{ij}} , \tag{S7}$$

where $a_{ij} = 1$ if countries i and j are connected and $a_{ij} = 0$ otherwise. Note that, in the above expression, a_{ij} depends on the real trade data, whereas $p(d_{h,ij})$ depends on the $\{(r, \theta)\}$ coordinates to be inferred. Moreover, the connection probability Eq. (S5) depends also on the global parameters N, $\langle k\rangle$, γ and β, so they must be known in order to compute (and therefore to maximize) Eq. (S7). The likelihood maximization thus requires the following two steps:

- **Estimating the global parameters:** The value of the exponent γ can be found from the linear fit to the curve $\log P_c(k)$ vs. $\log k$, where $P_c(k) = \int_k^\infty P(k')dk'$ is the complementary cumulative degree distribution giving the probability that a randomly chosen node in the network has degree greater or equal to k. From its expression, it is clear that for a power-law degree distribution with exponent γ, $P_c(k) \sim k^{1-\gamma}$. Thus, the curve $\log P_c(k)$ vs. $\log k$ is the straight line $\log P_c(k) = (1 - \gamma) \log k + c$, which allows to obtain γ from its slope. Due to the small number of nodes in the system, we have constrained the values of $\gamma \geq 2.15$.

  Although the values of the parameters N and $\langle k\rangle$ seem to be clear, they must be chosen carefully. When a synthetic network is generated with the model, some of its nodes may have no connections (which is more likely to occur for very sparse graphs) and we do not consider them as belonging to the system. Hence, the target N and $\langle k\rangle$ can be different from the observed ones. For very large systems, these differences can be estimated analytically, but in this case we are considering networks with less than 200 nodes.

  The value of β is also difficult to estimate. It affects the resulting average clustering coefficient $\bar{C}$ (indeed, $\bar{C}$ increases with increasing β) but the exact value cannot be obtained by analytic means, so we must devise some other method to compute its value.

  Due to theses difficulties, we have estimated the three parameters N, $\langle k\rangle$ and β by comparing the real networks to synthetic networks generated with the model using different values for the three target parameters. The detailed procedure we followed reads:

  1. Measure γ, N_{obs}, $\langle k\rangle_{obs}$ and $\bar{C}_{obs}$, i. e. the exponent of the degree distribution, the number of nodes, average degree and average clustering of the network.
  2. Initialize the target variables N_{targ}, $\langle k\rangle_{targ}$ and β_{targ} using some values as initial guess. In our case, we used $N_{targ} = N_{obs} + 10$, $\langle k\rangle_{targ} = 4\langle k\rangle_{obs}$ and $\beta_{targ} = 5.5$.
  3. Generate a synthetic network using the target variables and the observed value of γ.
  4. Measure N_{synth}, $\langle k\rangle_{synth}$ and $\bar{C}_{synth}$ on the synthetic network.
  5. If $|N_{synth} - N_{obs}| < \epsilon_N \wedge |\langle k\rangle_{synth} - \langle k\rangle_{obs}| < \epsilon_{\langle k\rangle} \wedge |\bar{C}_{synth} - \bar{C}_{obs}| < \epsilon_{\bar{C}}$, the synthetic network can be considered similar to the observed one, so the values N_{targ}, $\langle k\rangle_{targ}$ and β_{targ} used to generate it are good estimations to be used in Eq. (S7). Keep them and stop. If the condition is not fulfilled, go to step 6.
  6. If $N_{synth} < N_{obs} \Rightarrow N_{targ} + \delta_N \rightarrow N_{targ}$. Else $N_{targ} - \delta_N \rightarrow N_{targ}$.
     If $\langle k\rangle_{synth} < \langle k\rangle_{obs} \Rightarrow \langle k\rangle_{targ} + \delta_{\langle k\rangle} \rightarrow \langle k\rangle_{targ}$. Else $\langle k\rangle_{targ} - \delta_{\langle k\rangle} \rightarrow \langle k\rangle_{targ}$.
     If $\bar{C}_{synth} < \bar{C}_{obs} \Rightarrow \beta_{targ} + \delta_\beta \rightarrow \beta_{targ}$. Else $\beta_{targ} - \delta_\beta \rightarrow \beta_{targ}$.
     Go to step 3.

  Using this method, the resulting synthetic networks can be considered similar to the real ones, so the obtained values of the parameters are good estimations. In our case, for every World Trade Network, that is, for every network representing the trade of a particular year, we have run the algorithm explained above 20 times, so the values of N, $\langle k\rangle$ and β used in this work are an average over 20 estimations. Moreover, we have set $\epsilon_N = 1$, $\epsilon_{\langle k\rangle} = 0.1\langle k\rangle_{obs}$ and $\epsilon_{\bar{C}} = 0.05$. δ_N was set to 1, while $\delta_{\langle k\rangle}$ and δ_β had no fixed values; every time step 6 was run, their values were withdrawn from the uniform distribution $U(0, 0.2)$. To achieve the convergence of the method, it was also useful to constrain the value of β between 2.1 and 6.5.

- **Finding the coordinates of nodes:** Once the global parameters are known, Eq. (S7) can be maximized. To this end, we have used the Metropolis-Hastings algorithm with few variations, as explained below. Since we are not embedding a single network but a sequence of them, we have taken as initial coordinates of a given country

the ones obtained in the previous embedding (if the country belonged to it). Furthermore, we first embedded the network with more nodes, the one corresponding to year 2013, and the rest of them sequentially in decreasing order. Notice also that it is convenient to maximize the logarithm of L, which we will denote as log-likelihood $\ln L$, instead of L itself to avoid numerical issues. The steps to embed the network corresponding to year Y are:

1. **Initial positions:** for every country i in the current network:
   i. If i belongs to the WTW of year $Y+1$, set (r_i, θ_i) to the values of the $Y+1$ embedding (constrain r_i so that it is not larger than R). Else, choose r_i randomly from $U(0, R)$ and θ_i from $U(0, 2\pi)$.
   ii. Set $(\tilde{r}_i, \tilde{\theta}_i) = (r_i, \theta_i)$. These variables will record the maximum-likelihood configuration.
2. **Compute initial log-likelihood** $\ln L$ using Eq. (S7). Set $\ln \tilde{L} = \ln L$. This variable will record the highest value of the log-likelihood.
3. **Maximize likelihood:** Repeat $S \times N^2$ times (in all cases, we observed that $S = 30$ is enough to achieve convergence):
   i. Choose a node i at random from the list $[2, N]$; notice that we never select node 1 (which corresponds to the USA, the most persistent hub in time) in order to keep its angular coordinate unchanged. The reason to do so is that the likelihood L is invariant with respect to global rotations of the system. Hence, not varying the angular coordinate of one of the nodes anchors the embedding by breaking this symmetry.
   Propose a value for θ_i' from $U(0, 2\pi)$.
   Compute the log-likelihood increment as $\Delta \ln L = \sum_{j \neq i} a_{ij} \left(\ln p(d_{h,ij}') - \ln p(d_{h,ij}) \right) + (1 - a_{ij}) \left(\ln(1 - p(d_{h,ij}')) - \ln(1 - p(d_{h,ij})) \right)$. Notice that only $N-1$ steps are required, as opposed to calculating the new log-likelihood as in step 2.
   If $\Delta \ln L \geq 0$ set $\theta_i = \theta_i'$ to accept the change. Otherwise, accept the change with probability $e^{\Delta \ln L} = L_{new}/L_{old} < 1$.
   If the change is accepted, give $\ln L$ the new value $\ln L + \Delta \ln L \to \ln L$. If $\ln L > \ln \tilde{L}$ set $(\tilde{r}_i, \tilde{\theta}_i) = (r_i, \theta_i)$ and $\ln \tilde{L} = \ln L$.
   ii. Choose a node i at random from the list $[1, N]$.
   Propose a value for r_i' from $U(0, R)$.
   Compute the log-likelihood increment as $\Delta \ln L = \sum_{j \neq i} a_{ij} \left(\ln p(d_{h,ij}') - \ln p(d_{h,ij}) \right) + (1 - a_{ij}) \left(\ln(1 - p(d_{h,ij}')) - \ln(1 - p(d_{h,ij})) \right)$.
   If $\Delta \ln L \geq 0$ set $r_i = r_i'$ to accept the change. Otherwise, accept the change with probability $e^{\Delta \ln L} = L_{new}/L_{old} < 1$.
   If the change is accepted, give $\ln L$ the new value $\ln L + \Delta \ln L \to \ln L$. If $\ln L > \ln \tilde{L}$ set $(\tilde{r}_i, \tilde{\theta}_i) = (r_i, \theta_i)$ and $\ln \tilde{L} = \ln L$.

For every year Y, we have repeated this algorithm 10 times and kept the maximum-likelihood configuration in all 10 embeddings. We have then run step 3 starting with such initial configuration but accepting the changes if and only if $\Delta \ln L > 0$.

It is also important to point out that L has a very complex landscape with many degeneracies, that is, configurations with similar likelihood. Usually, the difference between these configurations is the inversion of a whole community. Such a change mildly affects a small fraction of the pairs of distances $d_{h,ij}$ only and, therefore, L takes a similar value. In order to obtain statistically reliable distances between countries, we have also found the coordinates of nodes in every network 100 times starting with completely random positions and computed their average distances (both hyperbolic and angular) and connection probabilities, as well as their fluctuations (see Fig. S6).

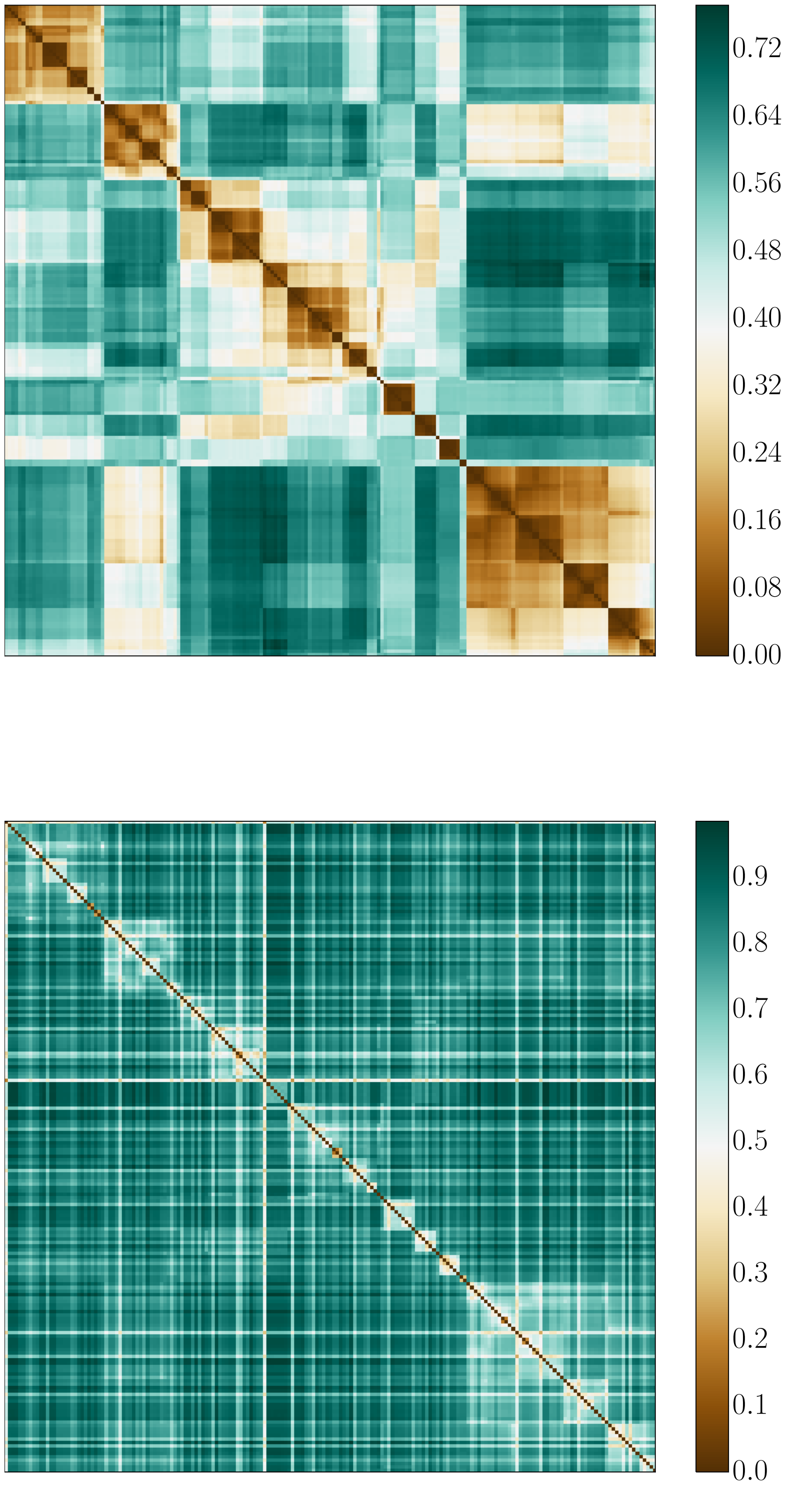

FIG. S6: **Top:** Average normalized angular distances $\langle \tilde{d}_{a,ij} \rangle$ between every pair of countries (i, j) in 2013. Nodes have been ordered such that nearest neighbours are consecutive. The dark squares in the matrix reveal the community structure of the WTW. **Bottom:** Average normalized hyperbolic distances $\langle \tilde{d}_{h,ij} \rangle$ between every pair of countries (i, j) in 2013. The ordering of the countries is the same as in the top matrix.

In order to quantify the quality of the embeddings, we have run local and global tests. Local tests aim at quantifying the pairwise similarities between the model and the real data, while the global tests we have run provide evidence of the meaningfulness of the embeddings by showing that the geometrical information can be used to navigate the network efficiently.

- **Local tests:** The first check we have run is computing numerically the empirical connection probability for each embedding. To do so, we have performed a binning in M subintervals of the interval in which all the possible values of the effective distance $e^{\frac{1}{2}(d_{h,ij}-R)}$ lay and then counted, for every bin, the fraction of connected pairs out of all those whose effective distances fall into the bin. If the embedding method works properly and the network can be described by the hyperbolic model, the empirical connection probability should resemble the Fermi-Dirac probability distribution function (see Eq. (S5)). The results are shown in Fig. S7.

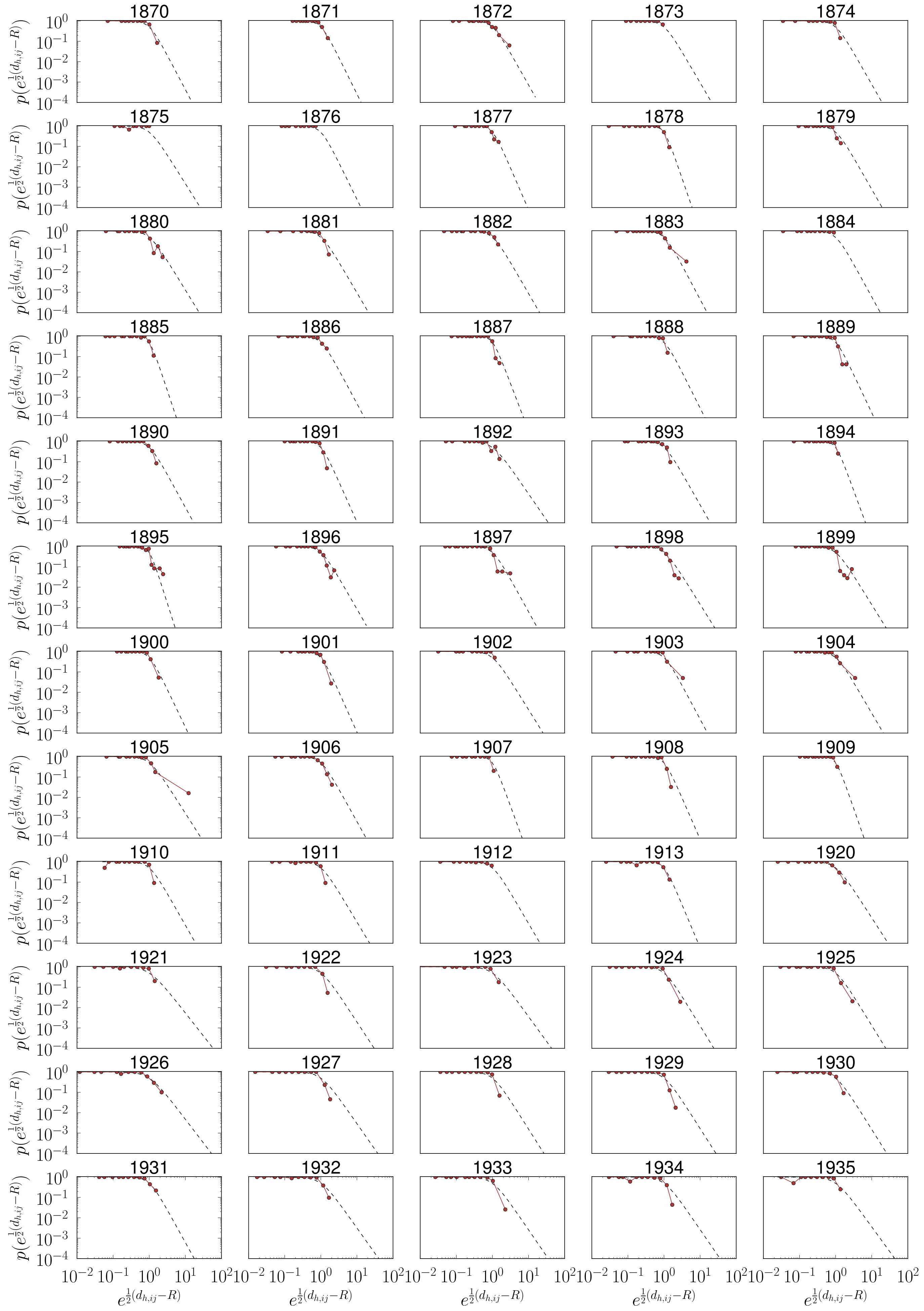
1870
1871
1872
1873
1874
1875
1876
1877
1878
1879
1880
1881
1882
1883
1884
1885
1886
1887
1888
1889
1890
1891
1892
1893
1894
1895
1896
1897
1898
1899
1900
1901
1902
1903
1904
1905
1906
1907
1908
1909
1910
1911
1912
1913
1920
1921
1922
1923
1924
1925
1926
1927
1928
1929
1930
1931
1932
1933
1934
1935
$p(e^{\frac{1}{2}(d_{h,ij}-R)})$
$e^{\frac{1}{2}(d_{h,ij}-R)}$
10^0
10^{-1}
10^{-2}
10^{-3}
10^{-4}
10^{1}
10^{2}

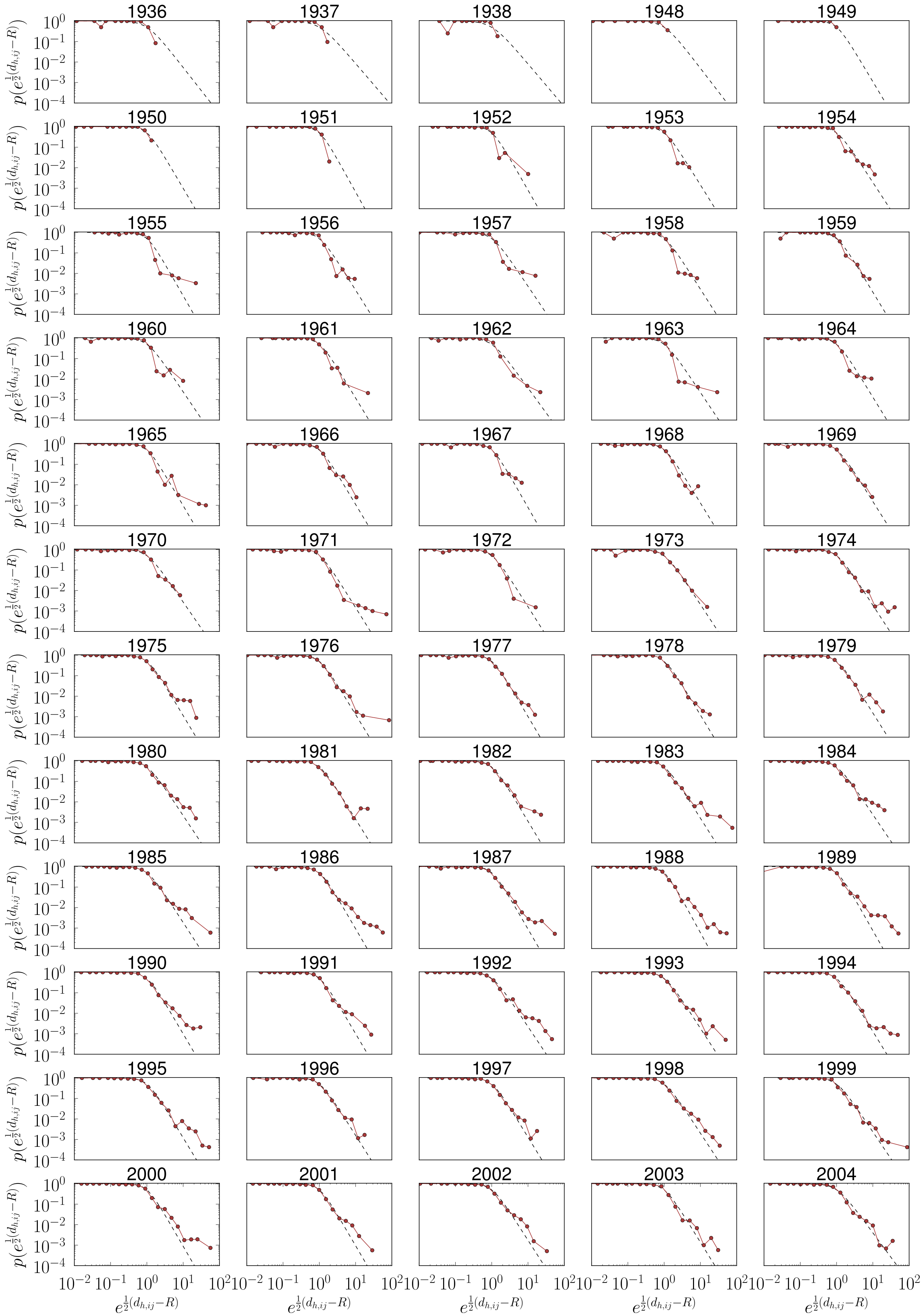
1936
1937
1938
1948
1949
1950
1951
1952
1953
1954
1955
1956
1957
1958
1959
1960
1961
1962
1963
1964
1965
1966
1967
1968
1969
1970
1971
1972
1973
1974
1975
1976
1977
1978
1979
1980
1981
1982
1983
1984
1985
1986
1987
1988
1989
1990
1991
1992
1993
1994
1995
1996
1997
1998
1999
2000
2001
2002
2003
2004
$p(e^{\frac{1}{2}(d_{h,ij}-R)})$
$e^{\frac{1}{2}(d_{h,ij}-R)}$

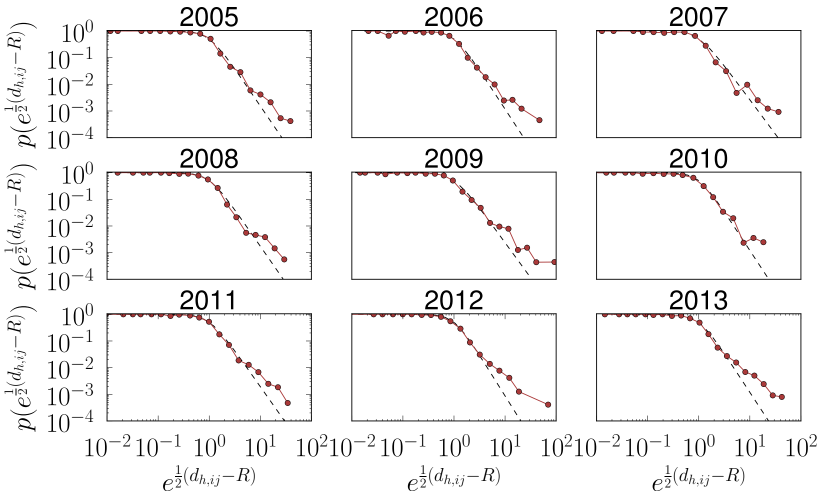

FIG. S7: Evolution of the empirical connection probability for all embeddings. The plots show a very good agreement between the theoretical curves (dashed lines) and the measurements in red, indicating both the similarity between the data and the model and the effectiveness of the embedding procedure described in Section II C.

The second local test we have considered is the receiver operating characteristic (ROC) curve. Once a network is embedded, Eq. (S5) assigns a connection probability p_{ij} to each pair of nodes, whether they are connected or not. If we define a threshold probability p, we can consider each pair as connected according to the model if and only if $p_{ij} > p$ and then compute the *true positive rate* (TPR)—the fraction of real links also predicted by the model—and the *false positive rate* (FPR)—the fraction of links that do not exist in the network but have been predicted to exist by the model. The result is a point in the TPR–FPR plane. Notice that a perfect prediction would correspond to TPR=1 and FPR=0. Since the test depends on the value of p, it is customary to compute the curve in the TPR–FPR space obtained by computing the two magnitudes for different values of p between 0 and 1. Fig. S8 shows the ROC curves for different years. Clearly, they approach the perfect prediction point for some value of p, evidencing the similarities between the WTW and the hyperbolic model. The area under the curve (AUC) provides a quantization of the results. The best possible result corresponds to AUC=1; as we show in the paper, its value is well above 0.975 in all embeddings.

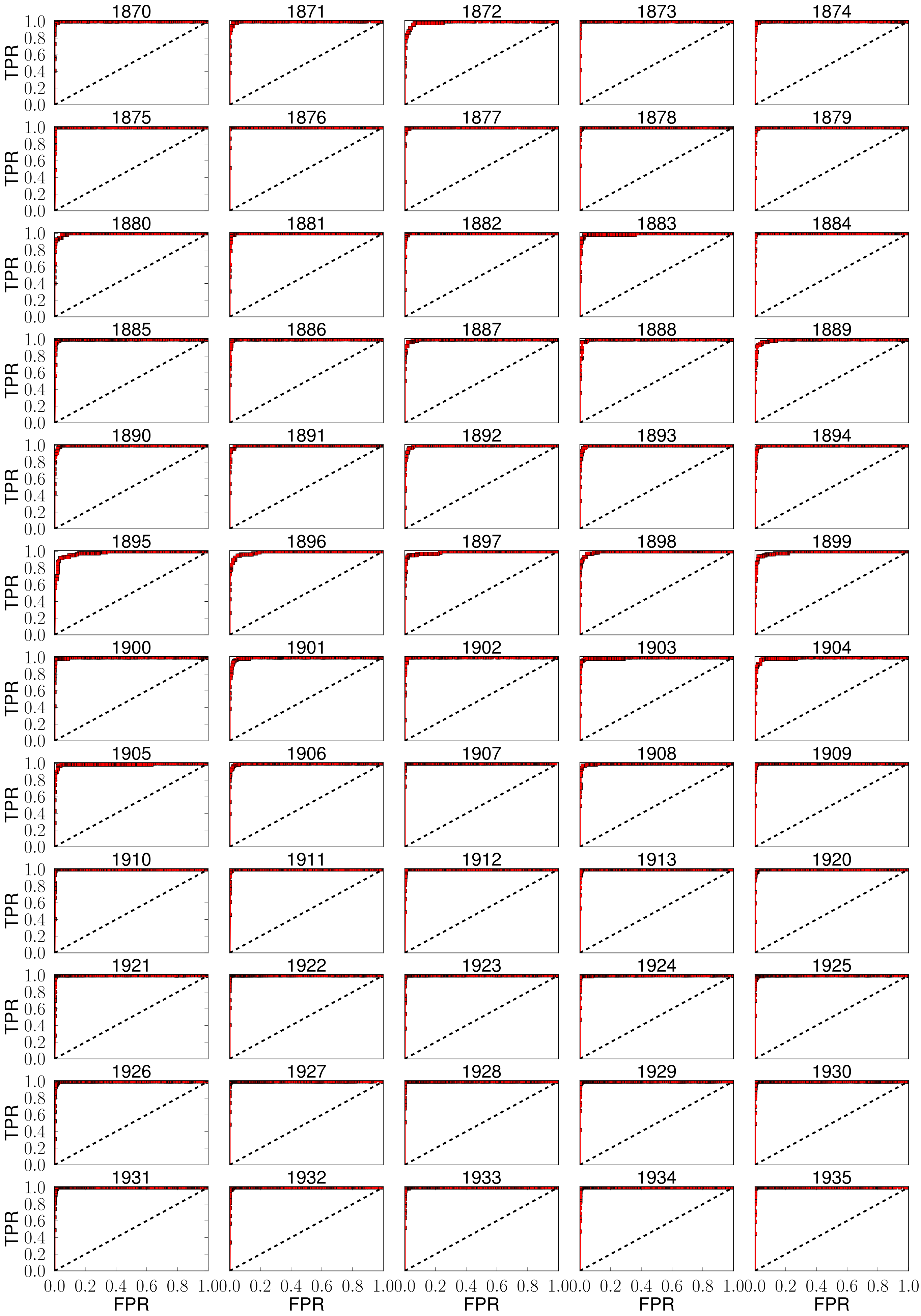

1870
1871
1872
1873
1874
1875
1876
1877
1878
1879
1880
1881
1882
1883
1884
1885
1886
1887
1888
1889
1890
1891
1892
1893
1894
1895
1896
1897
1898
1899
1900
1901
1902
1903
1904
1905
1906
1907
1908
1909
1910
1911
1912
1913
1920
1921
1922
1923
1924
1925
1926
1927
1928
1929
1930
1931
1932
1933
1934
1935
TPR
0.0 0.2 0.4 0.6 0.8 1.0
FPR
0.0 0.2 0.4 0.6 0.8 1.0

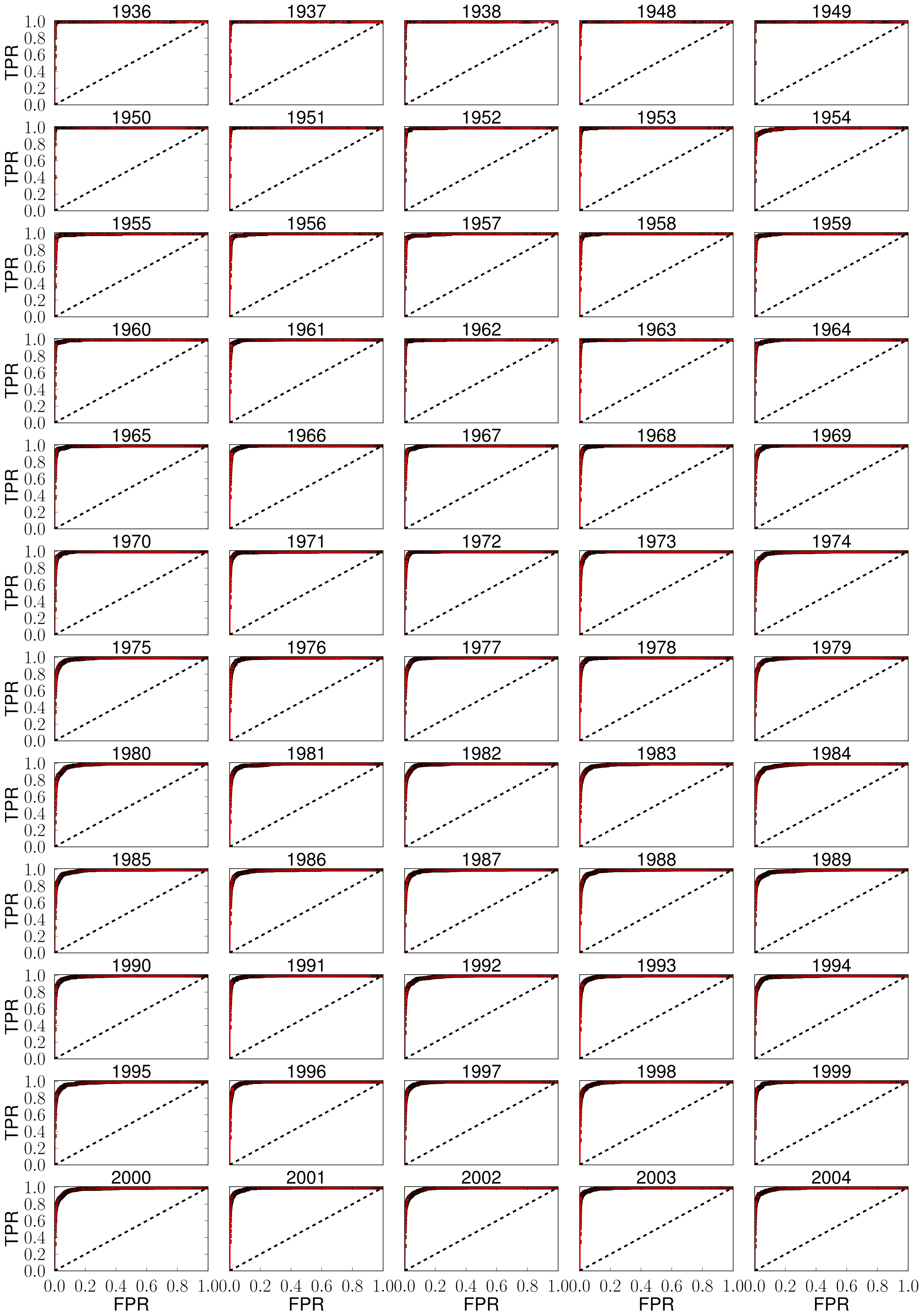
1936
1937
1938
1948
1949
1950
1951
1952
1953
1954
1955
1956
1957
1958
1959
1960
1961
1962
1963
1964
1965
1966
1967
1968
1969
1970
1971
1972
1973
1974
1975
1976
1977
1978
1979
1980
1981
1982
1983
1984
1985
1986
1987
1988
1989
1990
1991
1992
1993
1994
1995
1996
1997
1998
1999
2000
2001
2002
2003
2004
TPR
FPR
0.0 0.2 0.4 0.6 0.8 1.0

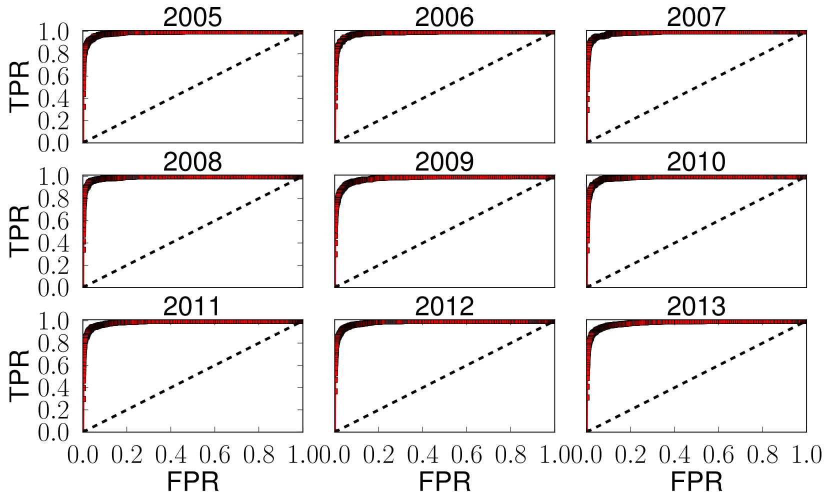

FIG. S8: Evolution of the reciever operating characteristic (ROC) curve, defined as the sequence of pairs (TPR, FPR) parametrized by the threshold probability p. All curves approach the optimal point TPR=1, FPR=0. Again, this evidences the good agreement between the data and the model. The integral of these curves is above 0.975 in all cases (see paper).

Finally, we have also computed the expected degree $\langle k_i \rangle$ of every node defined as $\langle k_i \rangle = \sum_{j \neq i} p_{ij}$, where p_{ij} is given by Eq. (S5) and the distance is calculated using the coordinates of the embedding and Eq. (S6). Fig. S9 shows that these quantities match almost perfectly the real values k_i, in accordance with MLE for expected degrees (Ref. [23] in the paper).

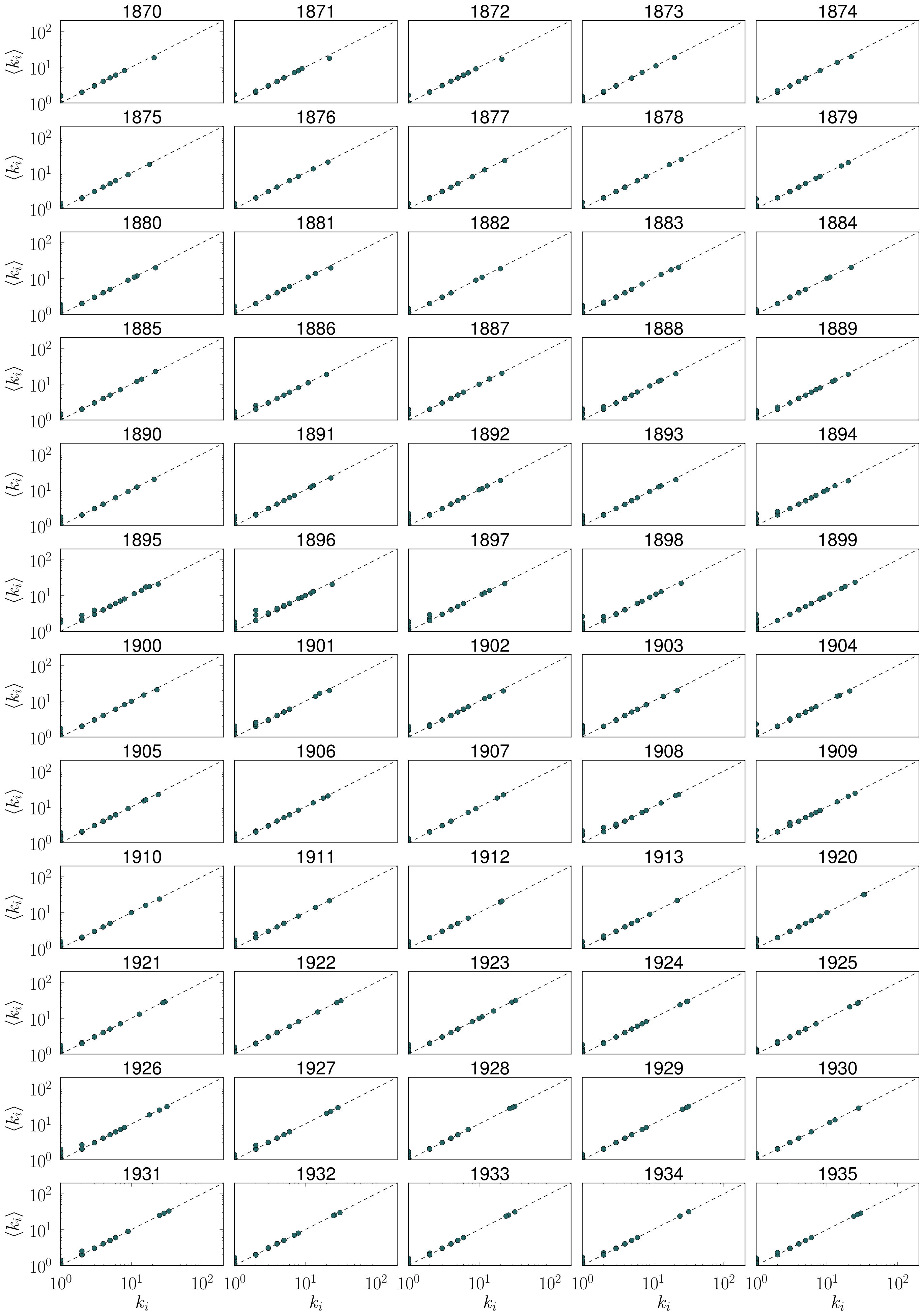
1870
1871
1872
1873
1874
1875
1876
1877
1878
1879
1880
1881
1882
1883
1884
1885
1886
1887
1888
1889
1890
1891
1892
1893
1894
1895
1896
1897
1898
1899
1900
1901
1902
1903
1904
1905
1906
1907
1908
1909
1910
1911
1912
1913
1920
1921
1922
1923
1924
1925
1926
1927
1928
1929
1930
1931
1932
1933
1934
1935
$\langle k_i \rangle$
10^0
10^1
10^2
k_i

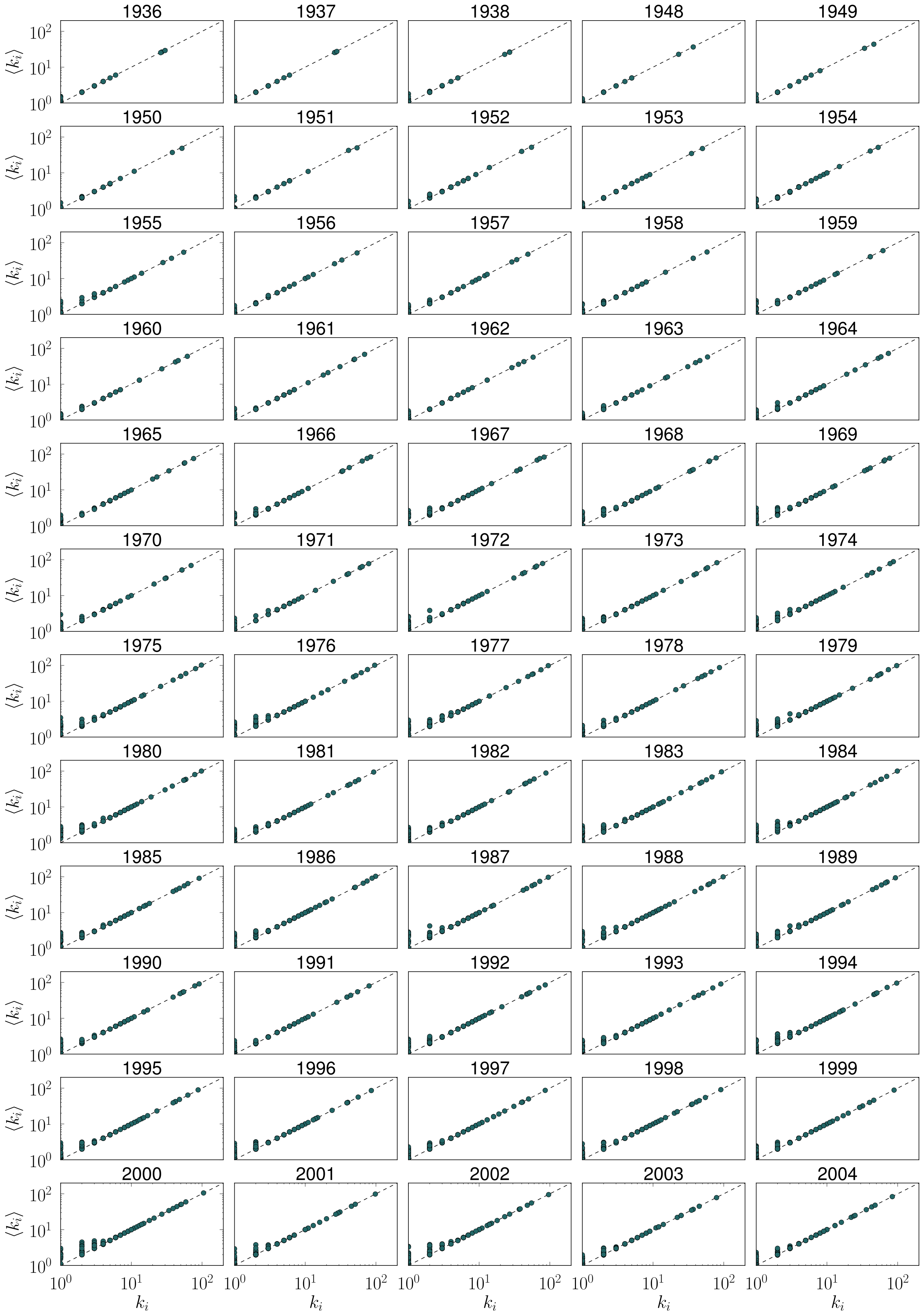
1936
1937
1938
1948
1949
1950
1951
1952
1953
1954
1955
1956
1957
1958
1959
1960
1961
1962
1963
1964
1965
1966
1967
1968
1969
1970
1971
1972
1973
1974
1975
1976
1977
1978
1979
1980
1981
1982
1983
1984
1985
1986
1987
1988
1989
1990
1991
1992
1993
1994
1995
1996
1997
1998
1999
2000
2001
2002
2003
2004
$\langle k_i \rangle$
k_i
10^0
10^1
10^2

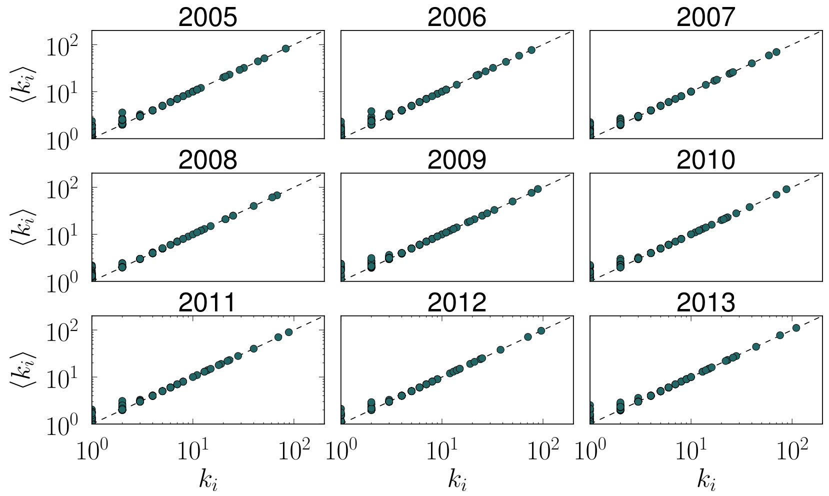

FIG. S9: Expected degrees of nodes $\langle k_i \rangle = \sum_{j \neq i} p_{ij}$ versus real degrees k_i. The two values are equal in almost all cases, which validates the quality of the description of the system by the model.

- **Global tests:** The three tests presented so far do only take into account local (pairwise) information. Hence, passing those tests is a necessary but not sufficient proof of the quality and meaningfulness of the geometrical information encoded by the embeddings. We now complement this validation by measuring the efficiency of the greedy forwarding procedure.

  Finding the shortest path between two nodes in a network is a computationally expensive problem. However, it has been previously shown (Ref. [23] in the paper) that the geometrical information provided by the embedding into hyperbolic space can be used to navigate the network efficiently by following the very simple rule of greedy forwarding: to go from any node to node j, hop to the current node's neighbour that is closest (according to Eq. (S6)) to j. Following this procedure in a badly embedded network can present two issues. On the one hand, one can get trapped describing loops endlessly and, on the other hand, even if the destination is reached the number of hops needed might be much larger than the shortest path length, i. e. the minimum number of hops actually required.

  We have thus measured the success rate—the fraction of pairs (i, j) for which it is possible to reach j from i via greedy forwarding—and the average stretch—the average over all successful routings of the quotient between the number of hops required by greedy forwarding and the shortest path length. Notice that, for every pair, both directions must be checked, since the process is not necessarily symmetric. We have also compared the results with the ones measured on the same networks with the same radial coordinates but with randomized angular coordinates. The results are displayed in Fig. S10. As the number of countries grows, the success rate for the randomized embeddings decreases, reaching values as low as 0.5, whereas it takes an almost constant value of 1 along the whole period for the real embeddings. The stretch increases for the last years in both cases. However, notice that it is always closer to 1 for the real embedding even though it is computed over almost all pairs of nodes, as opposed to the value corresponding to the randomized version, in which the computation only takes into account the successful fraction of pairs, which is low.

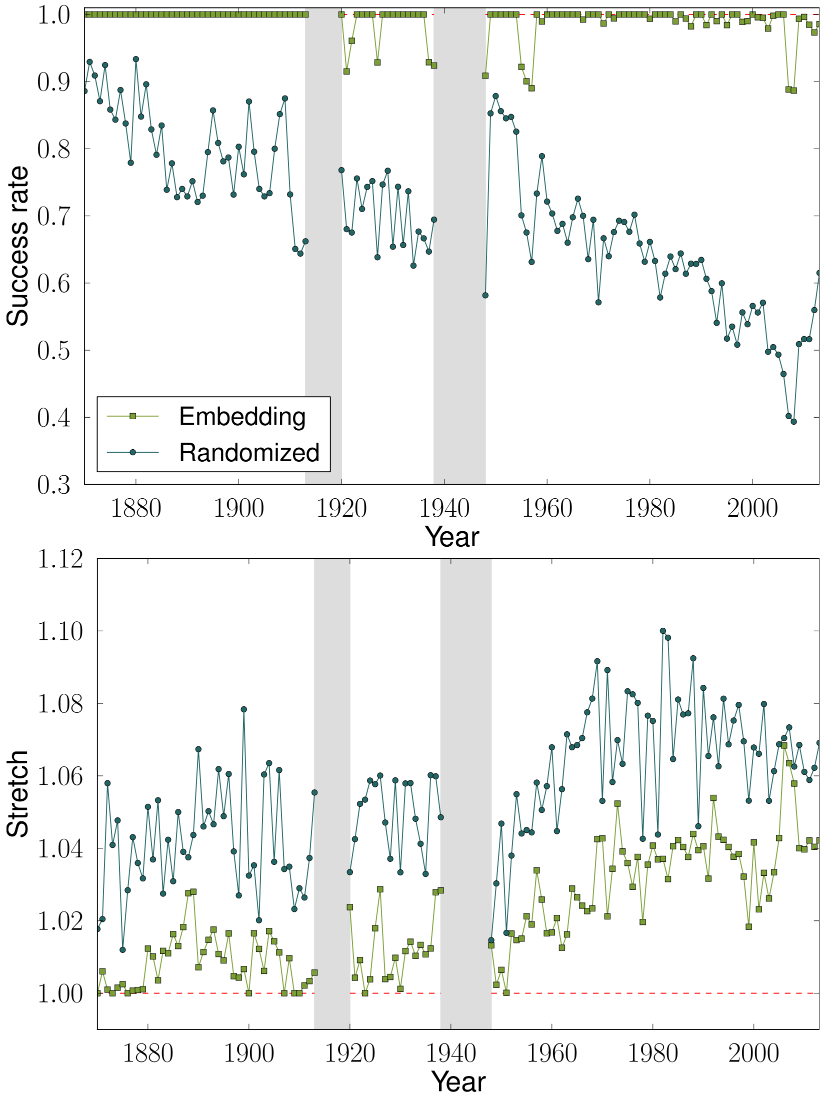

FIG. S10: Greedy forwarding metrics of the embeddings compared to the randomization of angular coordinates. In both cases, the real embedding values are closer to 1. **Top:** Success rate of greedy forwarding. **Bottom:** Stretch (average path length over shortest path length) of the successful greedy forwarding paths. Notice that the values for the real embeddings are lower even though they have been calculated over almost all possible pairs of nodes. Both results are strong evidence of the meaningfulness of the geometrical information contained in the embeddings; indeed, the coordinates of nodes are found using pairwise information only (Eq. (S7)), but the resulting map sucessfully encodes the global information required to efficiently navigate the network.

D. Community detection

Nodes near the center of the hyperbolic disk are close to many nodes in the system and therefore have a large number of connections, whereas nodes in the periphery are only close to those with a similar angular coordinate. According to Ref. [21] in the paper, the radial and angular coordinates can be interpreted as the popularity and similarity coordinates, respectively. Therefore, to partition the network into communities with similar nodes, we must take into account the angular coordinates of nodes. Indeed, the model assumes a uniform angular density, so the clusterization of nodes around certain angular coordinates reveals regions in which nodes tend to connect with each other profusely. The Critical Gap Method (CGM) relies in this idea to detect communities by finding angular regions that are densely populated in order to maximize modularity Q [9], the standard measure in community detection. This quantity, which is bounded $Q \in [-1, 1]$, compares the fraction of links inside communities with the expected fraction for a random distribution of edges, and is equal to 0 if nodes are randomly assigned into communities. The method then reads:

1. Set the value of $\tilde{Q} = -1$. This variable will encode the highest value of Q.

2. Increase the value of the critical gap g_c sequentially from 0 to 1. For every value:

    i. Connect all pairs of nodes (i, j) whose normalized angular distance $\tilde{d}_{a,ij} \leq g_c$.

    ii. Find all the connected components in the resulting graph.

    iii. Assign all nodes in the same connected component to the same community.

    iv. Compute the modularity Q of the partition. If $Q > \tilde{Q}$ set $Q \to \tilde{Q}$ and keep the partition.

At the end of the process, the partition with the highest modularity is kept. Notice that this method is equivalent to considering all gaps larger than g_c between consecutive nodes in the circle—that is, nodes between which there are no other nodes—as community boundaries. However, the algorithm we presented here is more general and allows working with distances instead of using coordinates. This is useful because average distances are more reliable but do not generally admit an embedding onto the circle (so they do not always contain information about consecutiveness). In Fig. S11 we compare the values of Q obtained using three methods: Louvain [10], Infomap [11] and CGM. Even though the modularity of the partitions obtained from CGM is slightly smaller than the one obtained from Louvain, the former show higher similarities with Preferential Trade Agreements.

In the paper we show the normalized mutual information (MI) between PTAs and both sets of partitions. The normalized mutual information of two partitions X and Y is defined as

$$MI = \frac{\sum\limits_{y \in Y} \sum\limits_{x \in X} p(x, y) \log \left(\frac{p(x,y)}{p(x)p(y)} \right)}{\max\{H(X), H(Y)\}}, \tag{S8}$$

where

$$H(X) = -\sum_{x \in X} p(x) \log p(x) \tag{S9}$$

and

$$H(Y) = -\sum_{y \in Y} p(y) \log p(y). \tag{S10}$$

Thus, if two partitions X and Y are equal, $p(x, y) = p(x) = p(y)$ and $MI = 1$. On the other hand, if the two partitions are uncorrelated, $p(x, y) = p(x)p(y)$, the logarithm in Eq. (S8) is null and, hence, $MI = 0$.

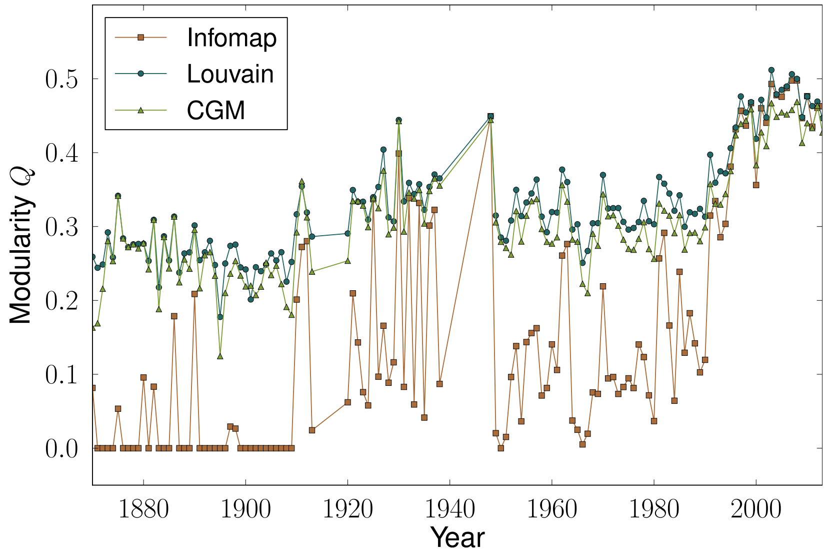

FIG. S11: Comparison between the modularities Q of the partitions found by three different methods. Both CGM and Louvain methods give better partitions than Infomap in almost all cases. The partitions found using CGM give modularities slightly lower than Louvain. However, the mutual information with PTAs is higher with the CGM partitions than with the Louvain partitions due to the capability of the former to resolve smaller communities.

E. Preferential Trade Agreements

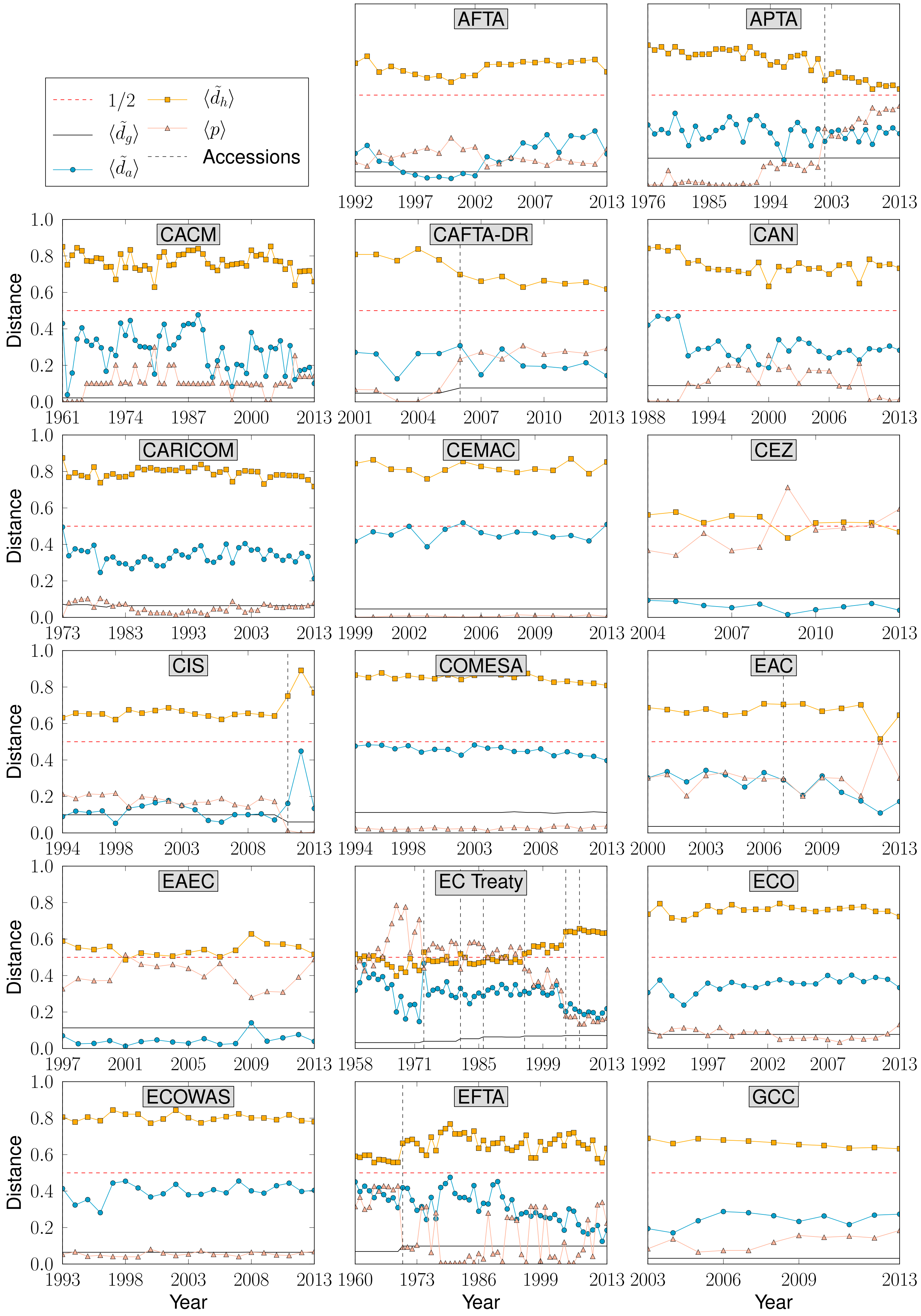

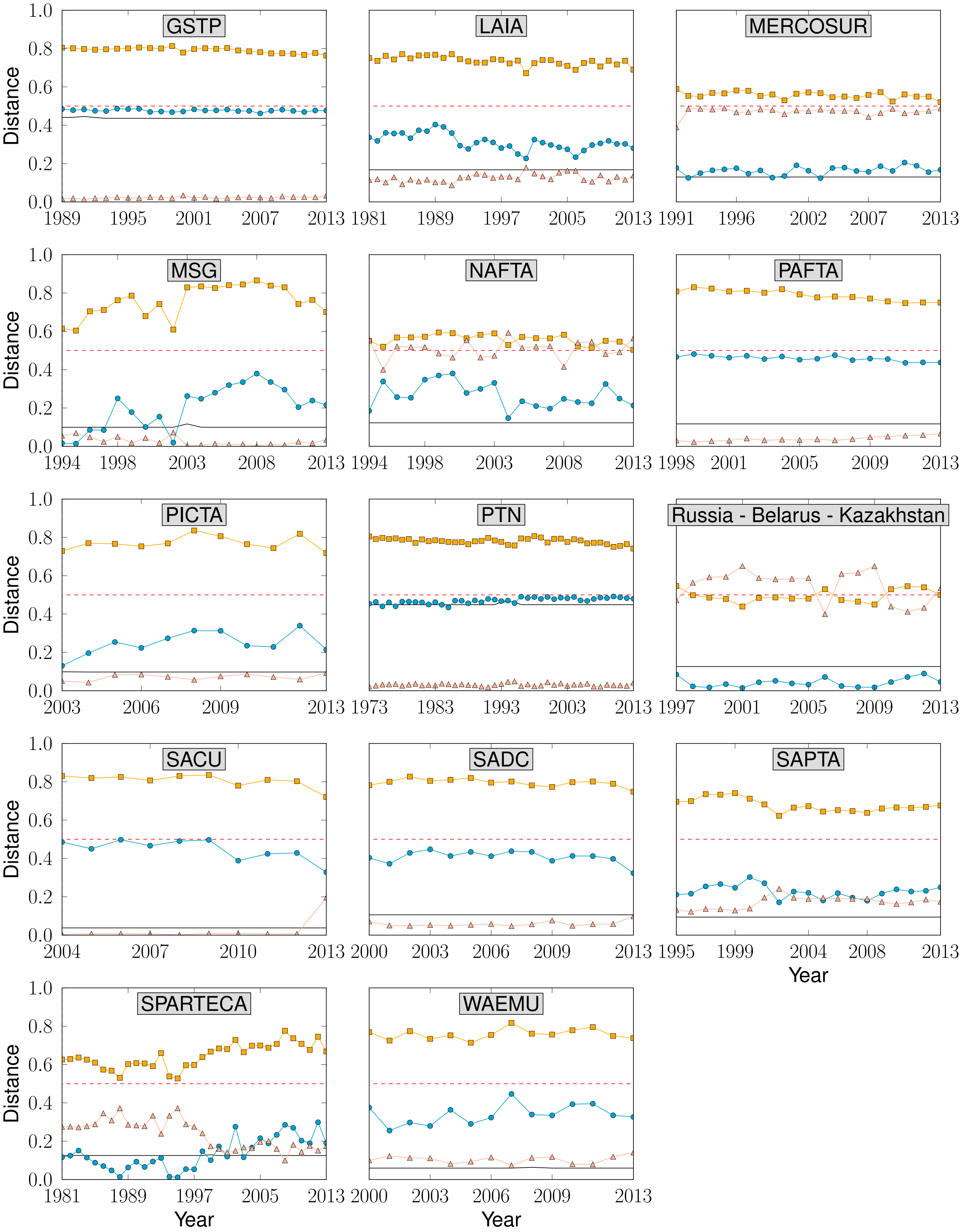

FIG. S12: Evolution of the average normalized distances (geographical $\tilde{d}_g$, angular $\tilde{d}_a$ and hyperbolic $\tilde{d}_h$) as well as the average connection probabilities between countries in every PTA. The vertical dashed lines represent the years of new accessions into the PTA. The red dashed lines correspond to the 1/2 value. Since distances are normalized with respect to the maximum possible distance in each case, it represents the average separation for both $\tilde{d}_g$ and $\tilde{d}_a$ for a uniform random distribution of points and the radius R in the hyperbolic case.

- **PTAs with $\langle \tilde{d}_a \rangle$ close but above the geographic value:** North American Free Trade Agreement NAFTA, Central American Common Market CACM, Caribbean Community and Common Market CARICOM, Pacific Island Countries Trade Agreement PICTA, Economic Cooperation Organization ECO, and PTAs in Africa, like SADC, WAEMU, CEMAC, ECOWAS, and in Latin America LAIA.

- **PTAs with $\langle \tilde{d}_a \rangle$ and $\langle \tilde{d}_g \rangle$ extremely congruent and a world-wide spread composition:** Protocol on Trade Negotiations PTN, Global System of Trade Preferences among Developing Countries GSTP.

- **PTAs with $\langle \tilde{d}_a \rangle$ and $\langle \tilde{d}_g \rangle$ extremely congruent and a strong geographical orientation:** Southern Common Market MERCOSUR, Commonwealth of Independent States CIS).

- **PTAs interconnecting Russia and the republics of the Soviet Union** (Russian Federation - Belarus - Kazakhstan, Eurasian Economic Community EAEC, Common Economic Zone CEZ) have a value of $\langle \tilde{d}_a \rangle$ below the geographical average.

- **PTAs for which both the average normalized angular and hyperbolic distances decrease, extremely mildly:** Pan-Arab Free Trade Area PAFTA, Dominican Republic - Central America - United States Free Trade Agreement CAFTA-DR, Andean Community CAN, Southern African Customs Union SACU, Common Market for Eastern and Southern Africa COMESA, East African Community EAC.

---

[1] International Monetary Fund, Direction of Trade Statistics (DOTS), `http://www.imf.org/en/Data`. (Date of access: 26/07/2016).

[2] The World Bank, `http://data.worldbank.org/`. (2016) (Date of access: 26/07/2016).

[3] ROC's Bureau of Foreign Trade, `http://www.trade.gov.tw/English/`. (2012) (Date of access: 26/07/2016).

[4] The Maddison-Project, `http://www.ggdc.net/maddison/maddison-project/home.htm`. (2013) (Date of access: 26/07/2016).

[5] Gleditsch, K. S., Expanded Trade and GDP Data, `http://privatewww.essex.ac.uk/~ksg/exptradegdp.html`. (Date of access: 26/07/2016).

[6] Central Intelligence Agency, The World Factbook, `https://www.cia.gov/library/publications/the-world-factbook/`. (Date of access: 26/07/2016).

[7] Johnston, L. and Williamson, S. H., What Was the U.S. GDP Then?, MeasuringWorth, `http://www.measuringworth.org/usgdp/`. (2016) (Date of access: 26/07/2016).

[8] Barthélemy, M, Gondran, B, and Guichard, E. (2003) Spatial structure of the internet traffic. *Physica A* **319**, 633–642.

[9] Newman, M. E. J. (2006) Modularity and community structure in networks. *Proc Natl Acad Sci USA* **103**, 8577–8582.

[10] Blondel, V. D, Guillaume, J.-L, Lambiotte, R, and Étienne Lefebvre. (2008) Fast unfolding of communities in large networks. *J Stat Mech* **2008**, P10008.

[11] Rosvall, M and Bergstrom, C. T. (2008) Maps of random walks on complex networks reveal community structure. *Proc Natl Acad Sci USA* **105**, 1118–1123.
